# Multifunctional Cationic Hyperbranched Polyaminoglycosides that Target Multiple Mediators for Severe Abdominal Trauma Management

**DOI:** 10.1002/advs.202305273

**Published:** 2023-11-23

**Authors:** Yongqiang Xiao, He Fang, Yuefei Zhu, Jie Zhou, Zhanzhan Dai, Hongxia Wang, Zhaofan Xia, Zhaoxu Tu, Kam W. Leong

**Affiliations:** ^1^ Department of Burn Surgery the First Affiliated Hospital Naval Medical University Shanghai 200433 P. R. China; ^2^ Department of Biomedical Engineering Columbia University New York NY 10027 USA; ^3^ ENT Institute Department of Facial Plastic and Reconstructive Surgery Eye & ENT Hospital Fudan University Shanghai 200031 P. R. China; ^4^ Department of Breast Surgery Affiliated Cancer Hospital and Institute Guangzhou Medical University Guangzhou 510095 P. R. China; ^5^ The Sixth Affiliated Hospital Sun Yat‐sen University Guangzhou Guangdong 510655 P. R. China; ^6^ Department of Systems Biology Columbia University Medical Center New York NY 10032 USA

**Keywords:** abdominal trauma, antibacterial properties, anticoagulation, biodegradable polyaminoglycoside, cfNA scavenging, inflammation modulation

## Abstract

Trauma and its associated complications, including dysregulated inflammatory responses, severe infection, and disseminated intravascular coagulation (DIC), continue to pose lethal threats worldwide. Following injury, cell‐free nucleic acids (cfNAs), categorized as damage‐associated molecular patterns (DAMPs), are released from dying or dead cells, triggering local and systemic inflammatory responses and coagulation abnormalities that worsen disease progression. Harnessing cfNA scavenging strategies with biomaterials has emerged as a promising approach for treating posttrauma systemic inflammation. In this study, the effectiveness of cationic hyperbranched polyaminoglycosides derived from tobramycin (HPT) and disulfide‐included HPT (ss‐HPT) in scavenging cfNAs to mitigate posttrauma inflammation and hypercoagulation is investigated. Both cationic polymers demonstrate the ability to suppress DAMP‐induced toll‐like receptor (TLR) activation, inflammatory cytokine secretion, and hypercoagulation by efficiently scavenging cfNAs. Additionally, HPT and ss‐HPT exhibit potent antibacterial efficacy attributed to the presence of tobramycin in their chemical composition. Furthermore, HPT and ss‐HPT exhibit favorable modulatory effects on inflammation and therapeutic outcomes in a cecal ligation puncture (CLP) mouse abdominal trauma model. Notably, in vivo studies reveal that ss‐HPT displayed high accumulation and retention in injured organs of traumatized mice while maintaining a higher biodegradation rate in healthy mice, contrasting with findings for HPT. Thus, functionalized ss‐HPT, a bioreducible polyaminoglycoside, holds promise as an effective option to enhance therapeutic outcomes for trauma patients by alleviating posttrauma inflammation and coagulation complications.

## Graphical abstract

This study documents the higher cell‐free nucleic acid (cfNA) levels in plasma from trauma patients and their correlation with inflammatory and coagulation indicators. Robust cfNA scavenging, superb antibacterial effects, and excellent anticoagulation efficacy make HPT and ss‐HPT satisfactory and ideal multifunctional biomaterials for the treatment of trauma and its associated complications. Moreover, the key therapeutic role of the cfNA scavenging strategy in the treatment of trauma is further verified.

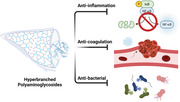

## 1 Introduction

Trauma is currently a worrying global public health issue.^[^ ^1^ ^]^ Based on reports by the World Health Organization (WHO), one person is injured every 6 s, and more than five million people die from trauma every year, accounting for ≈10% of all deaths worldwide, and the number of deaths due to trauma is 1.7 times higher than the number of deaths induced by malaria, tuberculosis, and HIV/AIDS combined.^[^ ^1^ ^]^ Trauma‐induced death could be linked to the excessive inflammatory response that occurs in patients, subsequent multiorgan failures, trauma‑induced coagulopathy (TIC), and posttrauma infection.^[^ ^2^ ^]^ Injured, hypoxic, and ischemic tissues expose the organism to damage‐associated molecular patterns (DAMPs), which may initiate a complex immunopathophysiological response that results in functional impairment.^[^ ^3^ ^]^

DAMPs, which are released from dying or dead cells of injured tissues and organs during trauma and are recognized by pattern recognition receptors (PRRs) of immune cells, could further trigger local and systemic inflammatory responses.^[^ ^4^ ^]^ Although the composition of DAMPs is extremely complicated, cell‐free nucleic acids (cfNAs), including cfDNA and cfRNA, are important categories that can induce the activation of several kinds of Toll‐like receptors (TLRs), such as TLR3, TLR8, and TLR9, thus activating the innate immune system and orchestrating the adaptive immune response.^[^ ^3^, ^5^ ^]^ In the case of severe abdominal trauma, this immune activation may trigger uncontrolled local or systemic inflammation and ultimately lead to fatal complications such as systemic inflammatory response syndrome (SIRS) and sepsis, in addition to multiple organ dysfunction syndrome (MODS).^[^ ^6^ ^]^

The clinical progression from SIRS to sepsis or septic shock has been recognized as being associated with an increased incidence of disseminated intravascular coagulation (DIC), which is characterized by widespread activation of coagulation leading to fibrin deposition in the vasculature, multiple organ failures, consumption of clotting factors and platelets, and life‐threatening hemorrhage leading to death.^[^ ^7^ ^]^ In addition to the inflammatory response that contributes to organ injury and multiple organ dysfunctions, cfNAs can activate coagulation factors XII and XI and cause platelet aggregation and fibrinolysis inhibition,^[^ ^8^ ^]^ thus inducing coagulation and thrombosis. However, overactivation of the coagulation system could result in the consumption of a large number of coagulation factors and platelets, ultimately contributing to DIC.^[^ ^9^ ^]^ Interestingly, some of the organ dysfunction associated with DIC is specific to severe trauma, which involves systemic coagulation and inflammatory activation. More importantly, the inflammatory response and clotting reaction can also reinforce each other, with inflammation leading to an accelerated coagulation cascade, while thrombosis also exacerbates the patients’ inflammatory response.^[^ ^7^, ^10^ ^]^

Based on the current knowledge, we propose that cfNA scavenging by nanomaterials could be a promising strategy to treat the dysregulated inflammatory response and hypercoagulation observed after trauma. As cfNAs are highly negatively charged, they could be electrostatically bound with cationic nanomaterials.^[^ ^11^ ^]^ Previously, a cfDNA scavenging strategy was successfully applied to treat several diseases involving excessive inflammation. For instance, cationic polyamidoamine dendrimer generation 3 (PAMAM‐G3) could be applied for effective pulmonary thromboembolism inhibition and treatment of carotid artery injury in mice.^[^ ^12^ ^]^ It was also proclaimed that cfNAs released from damaged or dead cells serve as critical autoantigens in rheumatoid arthritis (RA), thereby exacerbating the pathogenesis. cfNA‐scavenging nanoplatforms were generated to neutralize excessively produced cfNAs and modulate RA symptoms in a mouse model.^[^ ^13^ ^]^ In addition, cationic nanomaterials were employed for sepsis and inflammatory bowel disease (IBD) therapy via cfNA scavenging.^[^ ^14^ ^]^ All of these previous studies confirmed that the cfNA scavenging strategy could be applied for the treatment of cfNA‐associated inflammatory diseases, which opens a new opportunity for severe abdominal trauma management.

Beyond the dysregulated aseptic inflammatory response, bacterial infection is extremely challenging for trauma patients.^[^ ^15^ ^]^ The susceptibility of trauma patients to bacterial infection markedly increases due to severe bodily injury.^[^ ^16^ ^]^ Intra‐abdominal posttraumatic infection remains a major threat to life. In abdominal trauma, the probability of bacterial infection is greater due to intestinal injury reasons; therefore, the antibacterial properties should be considered when designing biomaterials for severe abdominal trauma management.^[^ ^17^ ^]^ Aminoglycoside antibiotics were widely used for bacterial infection, and tobramycin is one of the most commonly used aminoglycoside antibiotics.^[^ ^18^ ^]^ Additionally, the biocompatibility and biodegradability of nanomaterials are crucial for their further in vivo application and clinical translation.^[^ ^11c^ ^]^ Accordingly, it is of great importance to develop multifunctional biomaterials with anti‐inflammatory, anticoagulant, anti‐infection, and biodegradation properties that are biocompatible for posttrauma treatment. Although several commercial cationic polymers, such as PAMAM and poly(N,N‐dimethylacrylamide) (PDMA), have shown promising anti‐inflammatory therapeutic results by the cfNA scavenging strategy, high toxicity still impedes their further application.^[^ ^14b^ ^]^ In addition, these polymers lack intrinsic bactericidal ability, which is a considerable deficiency in current trauma management.^[^ ^11a^ ^]^ In our previous studies, we developed a novel cationic hyperbranched polyaminoglycoside by incorporating the antibiotic tobramycin into the polymeric structure. This polyaminoglycoside exhibited a high positive charge on its surface, owing to the presence of many amino groups,^[^ ^19^, ^20^ ^]^ which were utilized as crosslinking units in other applications.^[^ ^21^ ^]^ These nanomaterials possess very low toxicity and exhibit inherent bactericidal properties due to the antibiotics they contain. Additionally, a disulfide‐containing crosslinker could be applied to generate biodegradable hyperbranched polyaminoglycosides. Therefore, it is interesting and promising to explore the potential application of tobramycin‐based biodegradable polyaminoglycosides for the management of severe abdominal trauma through cfNA scavenging and anti‐infection treatment.

**Figure 1:**
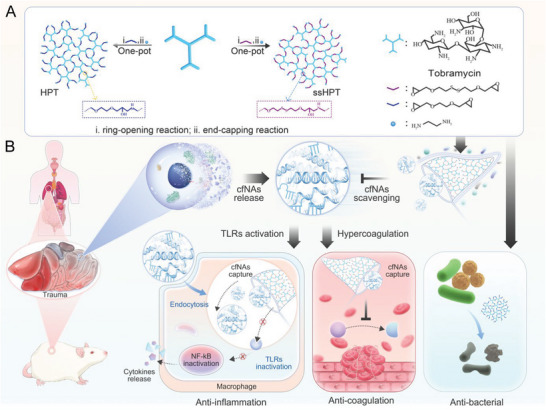
A) Synthesis of hyperbranched polyaminoglycosides HPT and ssHPT. B) Schematic illustration of cationic polyaminoglycosides used to treat mice subjected to abdominal trauma via multiple strategies.

In this work, we synthesized hyperbranched polyaminoglycosides embedded with tobramycin (HPT) and HPT‐containing disulfide linkages (ss‐HPT) and explored their applications in abdominal trauma treatment. The biocompatibility, biodegradability, and cfDNA binding ability of HPT and ss‐HPT were carefully investigated. Subsequently, we studied the anti‐inflammation, anticoagulation, and antibacterial properties of these polyaminoglycosides. Ultimately, the in vivo biodegradation of the materials and treatment of abdominal trauma with HPT and ss‐HPT were examined in the cecal ligation and puncture (CLP) mouse model (**Figure** **1**).

## 2 Results and Discussion

### 2.1 The Plasma cfNAs Level is Elevated in Trauma Patients

**Figure 2:**
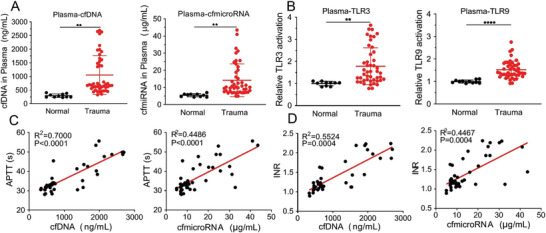
The relationship between cfNAs and features of trauma patients. A) The levels of cfDNA and cfmiRNA in plasma from healthy volunteers (*n* = 10) and trauma patients (*n* = 46). B) Relative TLR3 and TLR9 activation in plasma from healthy volunteers (*n* = 10) and trauma patients (*n* = 46). C) The correlation between the cfDNA, cfmiRNA, and APTT plasma levels of trauma patients. (*n* = 46) D) The correlation between the cfDNA, cfmiRNA, and INR plasma levels of trauma patients. (*n* = 46) Data represent the mean ± S. D. (^**^ *p* < 0.01, ^***^ *p* < 0.001).

To explore the changes in cfNAs in the plasma of trauma patients, 56 plasma samples, including samples from 46 trauma patients obtained within 24 h of injury and ten healthy volunteers, were collected. eight female patients and 38 male patients were included, and the mean age was 52.85 ± 15 years old. The concentrations of cfDNA and cfmiRNA in plasma were measured by corresponding testing kits. The results showed that the cfDNA and cfmiRNA levels in plasma were significantly higher in trauma patients than in healthy volunteers (**Figure** **2**A), which is consistent with previous reports.^[^ ^22^ ^]^ In addition, the cfDNA and cfmiRNA levels in plasma were proportionally correlated with the injury severity score (ISS) of trauma patients (Figure S2A,B, Supporting Information), meaning that the cfNA level was closely related to the severity of injury that occurred. Consistent with reports that cfNAs were agonists of several TLRs, plasma from trauma patients induced observably higher levels of TLR9 and TLR3 activation than that from controls (Figure 2B), triggering the subsequent immune cascade and causing uncontrolled inflammation‐related diseases, such as cytokine storms and multiorgan failure.^[^ ^4a,b^ ^]^ Procalcitonin (PCT) is another important indicator of the severity of the inflammatory response.^[^ ^23^ ^]^ We accordingly found that cfDNA and cfmiRNA levels were positively correlated with PCT levels (Figure S2C,D, Supporting Information), indicating a definite correlation between cfNAs and inflammation.

As previously mentioned, DIC is another serious posttrauma complication that can be characterized by the prolonged activated partial thromboplastin time (APTT), prothrombin time (PT), international normalized ratio (INR), decreased platelet (PLT) levels, and increased D‐dimer levels in plasma.^[^ ^7^ ^]^ The correlation between cfNA levels and coagulation indicators was also analyzed, and the results demonstrated that cfDNA and cfmiRNA levels were positively correlated with APTT, INR, and PT (Figure 2C,D; Figure S3A, Supporting Information). The PLT, which is involved in the coagulation mechanism, exhibited a negative correlation with cfDNA and cfmiRNA levels (Figure S3B, Supporting Information).

Based on these results, it can be concluded that increased cfNAs levels suggested prolonged clotting time, which seems to contradict the coagulation‐promoting effect of cfNA.^[^ ^8a^ ^]^ This finding indicated to us that cfNAs could induce DIC, during which diffuse activation of the clotting mechanism in the blood finally leads to the depletion of coagulation factors, leading to systemic bleeding tendencies.^[^ ^9b^ ^]^ Trauma patients are at a high risk of bleeding early after the injury; nevertheless, studies have also shown that the procoagulant mechanism and thrombogenic potential of trauma patients were significantly increased,^[^ ^9a^ ^]^ which were closely related to the severity of injury in patients^[^ ^9b^ ^]^ and became an independent risk factor for thrombosis in patients with severe trauma later.^[^ ^24^ ^]^ All these findings suggested that the elevated cfNAs levels in trauma patients indicated coagulation disorders and a dysregulated inflammatory state in trauma patients.

### 2.2 The Synthesis and Characterization of HPT and ss‐HPT

As cfNAs play a pivotal role in the inflammatory response and coagulation disorder in trauma patients,^[^ ^7a^ ^]^ it is interesting to develop a cfNA scavenging strategy with biomaterials to ameliorate undesired cfNA‐induced effects. Hyperbranched polyaminoglycosides were reported to have excellent biocompatibility and intrinsic antibacterial properties due to the inclusion of the antibiotic tobramycin in the chemical structure.^[^ ^21^ ^]^ In this work, HPT and ss‐HPT were prepared to assess their modulatory effects on inflammation, anticoagulation effects, and antibacterial properties in trauma patients. HPT and ss‐HPT were synthesized by a previously published method,^[^ ^21^ ^]^ and the detailed synthesis route is displayed in (Page S13 and Scheme S1, Supporting Information).

**Figure 3:**
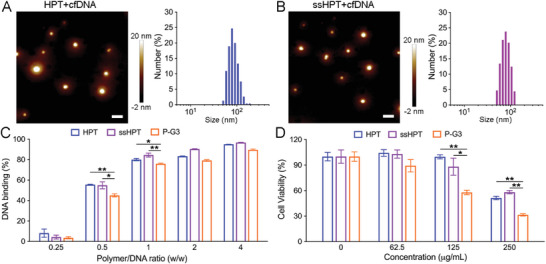
cfDNA binding and biocompatibility of HPT and ss‐HPT. AFM and DLS results of A) HPT+cfDNA and B) ss‐HPT+cfDNA analyses. Scale bars, 200 nm. cfDNA was incubated with HPT and ss‐HPT at 37 °C for 30 min. C) cfDNA binding efficacy of HPT, ss‐HPT, and P‐G3 with a series of ratios. data indicate the mean ±  S. D. (*n* = 3). D) Cell viability of HDFs after 24 h of treatment with HPT, ss‐HPT, and P‐G3 at a series of concentrations. Cells without any treatment were used as a negative control. Data indicate the mean ± S. D. (*n* = 3).

The molecular weights (Mw) of HPT and ss‐HPT were ≈1 × 10^4^ according to our previous work,^[^ ^21^ ^]^ and these polymers could not be observed by atomic force microscopy (AFM) due to their small size. Interestingly, AFM and dynamic light scattering (DLS) showed that the size of HPT and ss‐HPT increased to 70–80 nm after incubation with cfDNA (**Figure** **3**A,B), indicating successful cfDNA binding with polyaminoglycosides and the formation of nanoparticles. Herein, the DNA binding affinities of HPT and ss‐HPT were quantitatively tested using the PicoGreen kit, and the commercial cationic polymer PAMAM G3 (P‐G3) was applied as a control. The results revealed that both HPT and ss‐HPT demonstrated robust DNA binding affinity, which was higher than that of P‐G3, especially at polymer/DNA ratios of 0.5 and 1 (Figure 3C). However, the cytotoxicity of HPT and ss‐HPT is markedly lower than that of P‐G3 in human dermal fibroblast (HDF) cells (Figure 3D), which confirms their considerable potential biomedical application as cfDNA scavenging agents.

Unlike in HPT, a disulfide linkage was included in the ss‐HPT skeleton, which may provide ss‐HPT with responsiveness to reducing conditions. The quantitative degradation experiments (Figure S5, Supporting Information) also confirmed the biodegradation of ss‐HPT in highly reducing solutions. HPT displayed nearly no degradation in the reducing solution (10  or 1 mm GSH) after 24 h of incubation. In contrast, ≈70% of ss‐HPT was degraded in 10 mm GSH solution, while only 15% was degraded after 24 h incubation in 1 mm GSH solution, indicating that ss‐HPT is relatively stable in minimally reducing solutions but rapidly degrades under highly reducing conditions. Since GSH levels are relatively lower in patients with sepsis than in healthy individuals,^[^ ^25^ ^]^ ss‐HPT is expected to be retained in patients with trauma for a long time and rapidly degraded and excreted after recovery.

### 2.3 HPT and ss‐HPT Reduced TLR Activation and Proinflammatory Cytokine Generation

**Figure 4:**
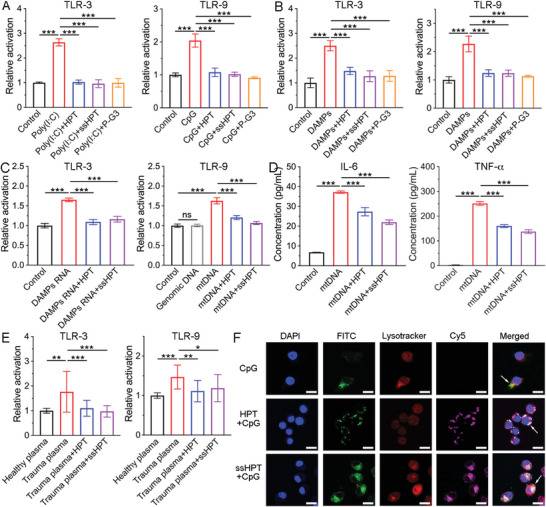
HPT, ss‐HPT, and P‐G3 reduced TLR activation and cytokine generation. A) Poly (I:C)‐induced TLR‐3 and CpG‐induced TLR‐9 activation in HEK‐TLR cells. (*n* = 3) B) DAMP‐induced TLR‐3 and TLR‐9 activation in HEK‐TLR cells. (*n* = 3) C) DAMP RNA‐induced TLR‐3 and mtDNA‐induced TLR‐9 activation in HEK‐TLR cells. (*n* = 3) D) DAMP‐induced IL‐6 and TNF‐α generation in THP1‐derived macrophage cells. (*n* = 3) E) Trauma plasma‐induced TLR‐3 and TLR‐9 activation in HEK‐TLR cells. (*n* = 19) F) CLSM images of RAW264.7 cells after 24 h incubation with CpG‐only, CpG+HPT, or CpG+ss‐HPT. CpG was labeled with FITC; HPT and ss‐HPT were labeled with Cy5; lysosomes were labeled with Lysotracker. Scale bars, 10 µm. Data represent the mean ± S. D. (*n* = 3, *p < 0.05, **p < 0.01, ***p < 0.001).

The dysregulated inflammatory response is one pathology observed in trauma patients.^[^ ^26^ ^]^ We already showed that cfNAs levels were markedly higher in the plasma of trauma patients than in the plasma of healthy volunteers, and cfNA‐induced TLR activation could result in an immune cascade and finally lead to serious inflammatory responses.^[^ ^3^, ^5^ ^]^ Interestingly, our results showed that HPT and ss‐HPT could suppress poly (I:C)‐induced TLR3 activation, ORN‐induced TLR8 activation, and CpG‐induced TLR9 activation in HEK Blue reporter cells (**Figure** **4**A; Figure S8A, Supporting Information). To simulate a practical trauma situation, HDF DAMPs were prepared by HDF cells after repeated freezing‐thawing cycles.^[^ ^27^ ^]^ Circulating mitochondrial DAMPs (MTDs), which have also been confirmed as a key link between trauma, inflammation, and SIRS, were also collected from isolated HDF mitochondria after repeated freeze‐thaw cycles.^[^ ^28^ ^]^ Further studies revealed that HDF DAMPs could induce TLR3, TLR8, and TLR9 activation and further induce the release of proinflammatory cytokines, such as IL‐1β and IL‐6 (Figures S6 and S7, Supporting Information). Excitingly, HPT and ss‐HPT effectively suppressed HDF DAMP‐induced TLR3, TLR8, and TLR9 activation (Figure 4B; Figure S8B, Supporting Information). Similarly, MTD also induced TLR9 activation, which was inhibited by HPT and ss‐HPT (Figure S10A,C, Supporting Information).

To further verify the mechanism of DAMP‐induced TLR activation, RNA, genomic DNA (gDNA), and mitochondrial DNA (mtDNA) were extracted from HDF DAMPs and MTDs using isolation kits. DAMP‐derived RNA activated TLR3 and induced the release of proinflammatory cytokines (Figure 4C; Figure S9, Supporting Information). However, mtDNA, which is unmethylated DNA, could activate TLR9 and induce cytokine release, while DAMP gDNA did not induce TLR9 activation (Figure 4C; Figure S10, Supporting Information). Similar to the previous poly (I:C) and CpG studies, DAMP RNA‐induced TLR3 activation and mtDNA‐induced TLR9 activation could be inhibited by HPT and ss‐HPT (Figure 4C). As the downstream signal of TLRs, CpG and mtDNA‐induced generation of inflammatory cytokines, such as TNF‐α and IL‐6,^[^ ^29^ ^]^ were also reduced by HPT and ss‐HPT, respectively (Figure 4D; Figure S11, Supporting Information). Consistent with the findings from HDF DAMPs, HPT or ss‐HPT treatment significantly suppressed plasma‐induced TLR3 and TLR9 activation in trauma patients (Figure 4E). Taken together, these findings indicate that cationic hyperbranched polyaminoglycosides HPT and ss‐HPT exhibited a considerable anti‐inflammatory effect through cfNA scavenging from DAMPs and the plasma of trauma patients.

It is interesting to elucidate the detailed mechanism of the anti‐inflammatory effect of HPT and ss‐HPT by the cfNA scavenging strategy. CpG DNA was applied to simulate cfNA in the following flow cytometry and confocal laser scanning microscopy (CLSM) measurements. Quantitative cellular uptake results obtained by flow cytometry revealed that HPT and ss‐HPT did not reduce the endocytosis of cfNAs by RAW264.7 macrophages (Figure S12, Supporting Information). However, previous results revealed that HPT and ss‐HPT effectively inhibited the activation of cfNA‐induced TLR activation. Therefore, we assume that the nanoparticles alter the interaction between cfNAs and TLRs, thereby attenuating the subsequent inflammatory cascade. To clarify this point, FITC‐labeled CpG and Cy5‐labeled HPT and ss‐HPT were added to Raw264.7 cells, and their intracellular colocalization was monitored over a 24 h period. Colocalization of Cy5‐labeled HPT, ss‐HPT, and FITC‐labeled CpG fluorescence was observed in endolysosomes (Figure 4F), which indicated that the internalization of HPT and ss‐HPT via endocytosis might be beneficial for blocking the recognition of CpG by macrophages.

### 2.4 HPT and ss‐HPT Modulate DAMP‐Induced Coagulation

**Figure 5:**
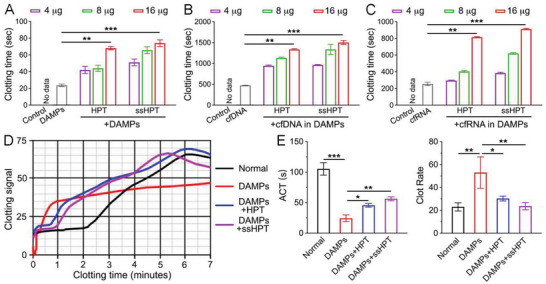
The anticoagulation properties of HPT and ss‐HPT. The clotting time of normal pooled human plasma after incubation with A) DAMPs, DAMPs+HPT, and DAMPs+ss‐HPT; B) DAMP‐derived cfDNA, cfDNA+HPT, and cfDNA+ss‐HPT; C) DAMP‐derived cfRNA, cfRNA+HPT, and cfRNA+ss‐HPT. D) The clotting signal of normal human whole blood after incubation with DAMPs, DAMPs+HPT, and DAMPs+ss‐HPT. E) ACT and clot rate of normal human whole blood after incubation with DAMPs, DAMPs+HPT, and DAMPs+ss‐HPT. The data were calculated by the clotting signal results in (D). Data represent the mean ± S. D. (*n* = 3, *p < 0.05, **p < 0.01, ***p < 0.001).

As mentioned previously, cfNA‐induced hypercoagulation may induce severe posttrauma DIC.^[^ ^7a^ ^]^ Therefore, it is important to examine whether HPT and ss‐HPT can alleviate cfNA‐induced coagulation. The inhibition efficacy of HPT and ss‐HPT in treating the kaolin‐based APTT but not PT was first confirmed using a standard APTT detection method and PT detection method (Figure S13A–F, Supporting Information). Then, the accelerated plasma clotting induced by DAMPs was confirmed to be dose‐dependent (Figure S14A, Supporting Information), while HPT or ss‐HPT treatment significantly attenuated DAMP‐induced plasma coagulation (**Figure** **5**A). To further determine the role of cfNAs in DAMPs during plasma coagulation, cfDNA, and cfRNA were isolated from the DAMPs, and cfNA‐related clotting tests were performed. Both DAMPs cfDNA and cfRNA could accelerate the clotting of human plasma (Figure S14B,C, Supporting Information), while the HPT or ss‐HPT treatments could significantly inhibit cfNA‐induced plasma coagulation via cfNA scavenging (Figure 5B,C).

Furthermore, a Sonoclot assay was applied to comprehensively evaluate the whole process of blood coagulation, activated clotting time (ACT), and clot rate (CR).^[^ ^30^ ^]^ ACT is the time a blood sample remains as a liquid until the beginning of fibrin formation.^[^ ^30^ ^]^ When the small aggregates of fibrinogen are converted to large fibrin aggregates, the blood viscosity changes, and the clotting signal is gradually strengthened as the gel develops.^[^ ^30^ ^]^ CR is the maximum slope of the Sonoclot Signature, which presents the coagulation conversion rate of fibrinogen to fibrin per unit of time.^[^ ^30^ ^]^ The results showed that DAMPs reduced the ACT of normal human whole blood from healthy volunteers from 105.3 ± 10.2 s to 24.3 ± 5.5 s and increased the CR from 23.0 ± 3.6 to 53.0 ± 13.8 sig min^−1^, suggesting that DAMPs could effectively activate clotting (Figure 5D,E). Consistent with the previous results, HPT or ss‐HPT treatment increased the ACT to 45.7 ± 3.1 s or 51.4 ± 4.6 s and decreased the CR to 30.3 ± 2.1 or 23.7 ± 3.2 s, respectively (Figure 5D,E). The findings above further indicate the detection of DAMP‐mediated hypercoagulability manifested as shortened ACT and increased CR values, while HPT or ss‐HPT could significantly inhibit DAMP‐induced hypercoagulability.

It was reported that cfNA in DAMPs could activate neutrophils to form neutrophil extracellular traps (NETs) (Figure S15A, Supporting Information),^[^ ^31^ ^]^ which is another factor that triggers coagulation.^[^ ^5^, ^32^ ^]^ Surprisingly, our results demonstrated that HPT or ss‐HPT treatment could also effectively abrogate NET‐induced plasma coagulation (Figure S15B, Supporting Information). These results showed that DAMPs, especially cfDNA and cfRNA, could promote plasma coagulation by activating clotting factors through the intrinsic coagulation pathway. Cationic hyperbranched polyaminoglycosides, including HPT and ss‐HPT, could suppress the DAMP‐induced intrinsic pathway of hypercoagulation through a cfNA scavenging strategy, protecting posttrauma DIC.

### 2.5 Antibacterial Activity of HPT and ss‐HPT

**Figure 6:**
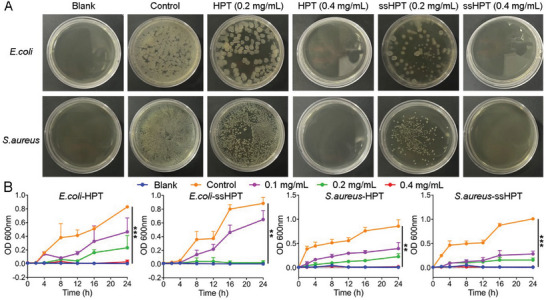
Antibacterial properties of HPT and ss‐HPT. A) Surface antibacterial activity of HPT and ss‐HPT against *E. coli* and *S. aureus* after 24 h of incubation at 37 °C. B) Growth curves of *E. coli* and *S. aureus* during a 24 h incubation with HPT and ss‐HPT. Data represent the mean ± S. D. (*n* = 3, ^**^ *p* < 0.01, ^***^ *p* < 0.001).

Considering the importance of antibacterial therapy in trauma treatment,^[^ ^15^ ^]^ the antibacterial properties of HPT and ss‐HPT against gram‐negative *Escherichia coli* (E. coli) and gram‐positive *Staphylococcus aureus* (S. aureus) were studied by measuring their growth curves. First, surface antibacterial experiments were performed by placing *E. coli* or *S. aureus* solutions with or without HPT and ss‐HPT onto the nutrient agar medium surface and incubating for 24 h. As shown in **Figure** **6**A, HPT or ss‐HPT (0.4 mg mL^−1^) displayed robust antibacterial ability due to the presence of antibacterial tobramycin in the chemical structure. The *OD*_600_ value of the bacterial solution was measured in the next step to calculate the bacterial growth curve. The results in Figure 6B show that the *OD*_600_ values obtained from *E. coli* and *S. aureus* samples without any treatments significantly increased after 4 h of incubation. Consistent with the previous results, HPT and ss‐HPT showed evident antibacterial activity in a concentration‐dependent manner. The number of bacteria incubated with HPT and ss‐HPT (0.4 mg mL^−1^) did not increase much after 24 h, indicating an almost 100% bacterial killing efficiency. The above results indicated that HPT and ss‐HPT exhibited favorable antibacterial activity against both *E. coli* and *S. aureus*, which is highly beneficial to infection prevention following severe abdominal trauma. While it is true that variations in material concentration exist between the antibacterial effects and clotting assays in our study, it is essential to note that both of these experiments were conducted in vitro. The effectiveness of the materials in vitro can be significantly influenced by factors such as bacterial load and duration of exposure. Given the distinct nature of these two in vitro experiments, it is reasonable to expect that the practical working concentration of the materials may differ. This disparity in concentration is a typical occurrence, as the optimal dosage for antibacterial effects may not necessarily align with that for clotting assays. The concentration variations were intentionally selected to achieve the desired outcomes in each specific assay.

### 2.6 Therapeutic Effect of HPT and ss‐HPT in the Abdominal Trauma Model

**Figure 7:**
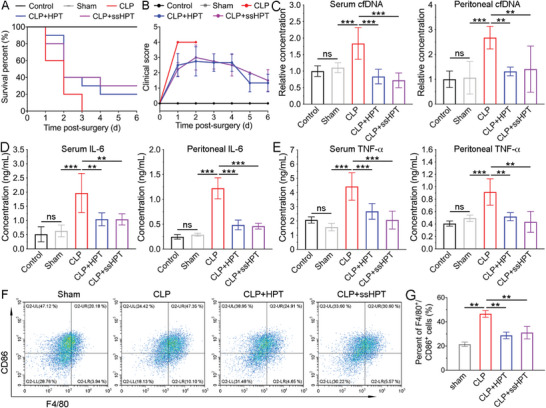
A) Survival rate and B) clinical score of mice in different treatment groups were recorded for 6 days after CLP. (*n* = 10) The levels of C) cfDNA, D) IL‐6, and E) TNF‐α were measured in and peritoneal fluid in different treatment groups at 24 h post‐CLP (*n* = 6). F) Flow cytometry and G) quantitative analysis of M1 macrophages in the peritoneal fluid of CLP mice in different treatment groups. Data represent the mean ± S. D. (*n* = 3, *p < 0.05, **p < 0.01, ***p < 0.001).

The experiments above confirmed that HPT and ss‐HPT could block cfNA‐induced inflammation, regulate cfNA‐induced hypercoagulation, and inhibit bacterial growth, which is of great importance for the management of clinical abdominal trauma. In the next step, an abdominal trauma mouse model was established by the CLP method^[^ ^14b^ ^]^ to explore the in vivo therapeutic effect of HPT and ss‐HPT. The survival rate, clinical score, and body weight of mice in different treatment groups were recorded for six consecutive days after the CLP operation. All animals in the untreated CLP group died within 72 h, while intraperitoneal (i.p.) administration of HPT (4 mg kg^−1^) and ss‐HPT (4 mg kg^−1^) in CLP mice produced survival rates of 20% and 30%, respectively, indicating that the treatment delayed trauma‐induced death (**Figure** **7**A). Furthermore, the clinical score was significantly reduced in the HPT‐ and ss‐HPT‐treated groups, indicating restoration of physical and mental states after treatment (Figure 7B), which was further validated by increased mouse body weight 3 days after HPT and ss‐HPT treatment (Figure S16, Supporting Information).

As shown in Figure 7C, both the cfDNA levels in serum and peritoneal fluid were dramatically higher in mice subjected to CLP operation than in the control and sham groups. Notably, HPT or ss‐HPT treatment almost completely reduced the cfDNA levels to normal levels. Consistent with findings regarding the cfDNA level, HPT or ss‐HPT also alleviated the elevated release of proinflammatory cytokines, including IL‐6 and TNF‐α (Figures 7D,E). In addition, flow cytometry analysis of peritoneal macrophages showed that HPT and ss‐HPT treatment reduced the percentage of proinflammatory M1‐polarized macrophages and increased the percentage of anti‐inflammatory M2‐polarized macrophages (Figure 7F,G; Figure S17, Supporting Information).

Although the 30% survival rate seems low, it is still meaningful because this model is severe in that all the mice in the CLP group died by day 3; the 20–30% survival rates in the HPT or ssHPT treatment groups are still significant and valuable.^[^ ^33^ ^]^ We also gave only one dose to the animals. If the dosing regimen is further optimized, which will be explored in future studies, the survival rate will likely improve.

**Figure 8:**
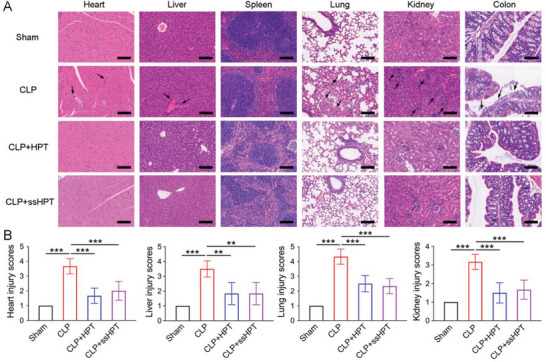
HPT and ss‐HPT attenuate multiple organ injury in CLP‐induced severe sepsis. A) H&E staining results of hearts, livers, spleens, lungs, kidneys, and colons from CLP mice in different treatment groups. Scale bars, 200 µm. Arrows indicate injury sites. B) Corresponding heart, liver, lung, and kidney injury scores were evaluated based on established criteria. Data represent the mean ± S. D. (*n* = 6, *p < 0.05, **p < 0.01, ***p < 0.001).

Posttrauma uncontrolled inflammatory responses and even cytokine storms may induce severe organ damage in patients.^[^ ^34^ ^]^ In this study, histopathological analysis was further performed to examine the effect of cationic polyaminoglycosides on major organ injury after trauma. Leukocyte infiltration and tissue injury were observed in multiple organs of CLP mice, including the lungs, kidneys, hearts, spleens, colons and livers (**Figure** **8**A). The organ injury scores were also evaluated (Figure 8B). Both the results confirmed that HPT and ss‐HPT treatment exhibited a protective effect on these organ injuries, indicating their great potential for the management of excessive inflammation after trauma.

### 2.7 Biodistribution, Biodegradation and Biosafety of HPT and ss‐HPT

**Figure 9:**
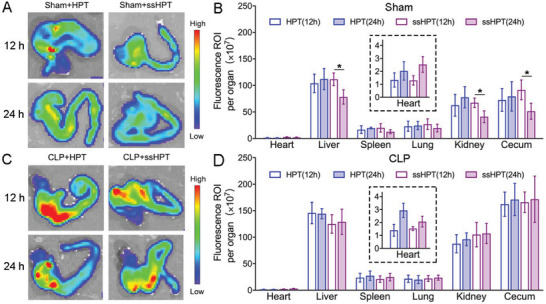
Biodistribution and biodegradation of HPT and ss‐HPT in sham mice and CLP mice. A) Ex vivo fluorescence images of ceca and B) quantitative assessment of therapy distribution in major organs of sham mice after 12 and 24 h treatment with HPT and ss‐HPT. C) Ex vivo fluorescence images of ceca and D) quantitative assessment of therapy distribution in major organs of CLP mice after 12 and 24 h treatment with HPT and ss‐HPT. Data represent the mean ± S. D. (n = 3, *p < 0.05).

The biodistribution and biodegradation of HPT and ss‐HPT were assessed by ex vivo fluorescence imaging in both sham and CLP mice. The cationic polyaminoglycosides were labeled with Cy5 and then intraperitoneally administered to sham and CLP mice. The major organs were harvested from both sham and CLP mice at 12 and 24 h after cfNA scavenger treatment, and the quantitative fluorescence intensity was calculated (**Figure** **9**). Both HPT and ss‐HPT were preferentially localized in the liver, kidney, and cecum in sham and CLP mice (Figure 9B,D). In addition, the level of accumulation was markedly higher, especially in the cecum in CLP mice, in inflammatory environments, which indicates that this strategy of cfNA scavenging is conducive to managing the excessive inflammation resulting from trauma. Interestingly, ss‐HPT exhibited a longer retention in the organs of CLP mice, while its fluorescence intensity decreased quickly in the liver, kidney, and cecum of sham mice (Figure 9B,D). As mentioned previously, increasing oxidative stress and declining glutathione levels in sepsis patients are much more obvious than in healthy people, and this situation is similar in CLP mice and sham mice.^[^ ^25^, ^35^, ^36^ ^]^

Therefore, the results suggested the biodegradability of ss‐HPT under normal conditions rather than in the environment of high oxidative stress associated with severe abdominal trauma conditions. This feature will be a remarkable benefit for future clinical translation if ss‐HPT is relatively stable in trauma patients while displaying accelerated biodegradation and excretion after the recovery of patients.

Subsequently, we determined the safety profile of HPT and ss‐HPT in healthy mice by evaluating the long‐term toxicity of these scavengers with high dosing (20 mg kg^−1^) for 7 and 14 days. Major organs, including the heart, liver, spleen, lung, and kidney, were harvested 7 and 28 days after administration of the nanomaterials. Histopathological sections treated with high doses of HPT for 28 days showed slight tissue damage. In contrast, ss‐HPT showed no obvious toxicity (Figures S23 and S24, Supporting Information), suggesting the safety of ss‐HPT for future clinical translation.

## 3 Conclusion

This study documented the higher cell‐free nucleic acid (cfNA) levels in plasma from trauma patients and their correlation with inflammatory and coagulation indicators. Cationic hyperbranched polyaminoglycosides, HPT, and ss‐HPT, containing tobramycin were developed to mitigate the trauma‐induced inflammation. Unlike HPT, ss‐HPT demonstrated gradual biodegradation under reducing conditions, facilitated by the disulfide component in its chemical structure. Both HPT and ss‐HPT exhibited low cytotoxicity, robust cfDNA binding efficiency, and efficient inhibition of cfNA‐ and DAMP‐induced inflammatory cascades and hypercoagulation in vitro. Additionally, they displayed excellent antibacterial properties attributed to their antibiotic composition. Crucially, in a cecal ligation puncture (CLP) mouse trauma model, HPT and ss‐HPT demonstrated excellent anti‐inflammatory effects and ameliorated multiple organ injuries. These findings indicate that ss‐HPT can serve as a “nanotrap,” efficiently scavenging circulating cfNAs, reducing inflammation, and halting the progression of associated trauma. Consequently, ss‐HPT offers a novel and promising treatment option for the management of severe abdominal trauma.

## 4 Experimental Section

### Clinical Sample

Blood samples from 46 trauma patients from the burn and trauma intensive care unit of Changhai Hospital were collected within 24 h of injury. Ten blood samples from healthy individuals were donated voluntarily by postgraduates and doctors of Changhai Hospital. The collection of blood samples was approved by the ethics committee of Changhai Hospital (CHEC2019‐150). All patients and healthy donors were informed of the purpose of the donated blood samples, and the consent and signatures of the patients and healthy donors were received. All blood samples were collected using EDTA anticoagulant tubes. After centrifugation at 3000 rpm for 10 min at 4 °C, the plasma was divided into sterile EP tubes and stored at −80 °C. The cfDNA and cfmiRNA concentrations of plasma were measured using the Quant‐iT PicoGreen dsDNA Kit and Quant‐iT microRNA Assay Kit, respectively, following the manufacturer's protocols. The trauma patients were also scored according to the detailed rules of injury grading (AIS score), and the final ISS score of the patients was equal to the sum of the squares of the highest AIS score of the three different sites.^[^ ^37^ ^]^ Ten microliters of plasma from trauma patients and normal donors were used to treat TLRs, and 5 µg mL^−1^ polymers were used to inhibit this activation. Coagulation data and inflammatory indicators were collected from trauma patients within 24 h of injury through the hospital's record management system, and this information included activated partial thromboplastin time (APTT), prothrombin time (PT), thrombin time (TT), international normalized ratio (INR), platelet count (PLT), D‐dimer levels, fibrin degradation product (FDP) levels, FIB (fibrinogen) levels, and procalcitonin (PCT) levels.

### Plasma Coagulation Assay

Human plasma coagulation assays were conducted according to a previous method with minor modifications.^[^ ^12^, ^38^ ^]^ HDF DAMP‐ or DAMP‐derived DNA and RNA with or without cationic materials were added to 50 µL normal pooled human plasma in sodium citrate (George King Bio‐Medical Inc., Overland Park, KS). The mixture was incubated for 3 min at 37 °C, followed by the addition of 50 µL of CaCl_2_ (25 mm). Clotting times were recorded using a STart Hemostasis Analyzer (Diagnostica Stago).

### Animal Study

All mice were cared for according to the instructions and approval of the Institutional Animal Care and Use Committee of The Sixth Affiliated Hospital, Sun Yat‐sen University (No. IACUC‐2022112801). Adult male C57BL/6 mice aged 6–8 weeks and weighing 20–25 g were fed, housed, and used in the ACS animal feeding system with 12–12 h light, free water, and food. To investigate the in vivo therapeutic effect of HPT and ss‐HPT, a mouse model of severe abdominal trauma, cecal ligation, and puncture (CLP) was established according to a previous report^[^ ^14b^ ^]^ Briefly, the mice were anesthetized with isoflurane and fixed to a board, the abdominal hair was shaved, and the abdomen was then disinfected with an alcohol disinfecting pad. A 1 cm incision was then made along the midline, and the cecum was gently removed from the abdominal cavity. The cecum was ligated using 4–0 silk at the designated site and punctured with a 21 G needle, and then cecal contents were extruded from the perforation. The cecum was gently placed back into the peritoneal cavity, and the incision was sutured and closed. The mice were divided into three groups and subjected to different treatments. Only abdominal incision and suture, but not cecal ligation and puncture, were performed in sham mice. The CLP group mice were given an intraperitoneal injection of 1 mL of normal saline to prevent immediate postoperative death. In the treatment groups, HPT (4 mg kg^−1^) or ss‐HPT (4 mg kg^−1^) in 1 mL normal saline was administered immediately after CLP, and the survival rate, clinical scores, and body weight were monitored for 6 days. Each group had ten mice.

### cfDNA and Inflammatory Cytokine Measurements in the Serum and Peritoneal Lavage Fluid

Twenty‐four hours later, the mice were sacrificed, blood was collected, 1 mL PBS was injected into the abdominal cavity of the mice, the abdomen of the mouse was gently rubbed several times, and peritoneal lavage fluid was collected. The cfDNA concentration was measured using the Quant‐iT PicoGreen dsDNA Assay Kit, and TNF‐α and IL‐6 concentrations were measured using the ELISA assay (Thermo Fisher Scientific), both in the serum and peritoneal lavage fluid. Major organs were harvested and fixed in 4% paraformaldehyde, embedded in paraffin, sectioned, and stained with hematoxylin and eosin for histological evaluation. Injury scores were determined as described previously.^[^ ^14b^ ^]^

### Ex Vivo Fluorescence Imaging

Mice were intraperitoneally injected with Cy 5‐labeled HPT and ss‐HPT at a dose of 4 mg kg^−1^ post‐CLP. The major organs, including the heart, lung, liver, spleen, kidney, and cecum, were excised and imaged at 12 and 24 h post‐CLP.

### The Ratio of M1‐Polarized Macrophages in the Peritoneal Cavity

Flow cytometry was used to determine the proportion of M1‐polarized macrophages in the abdominal cavity. The cells were collected by peritoneal lavage with 1 mL PBS containing 10% fetal bovine serum, centrifuged, and resuspended in PBS. Anti‐CD86‐FITC, anti‐CD206‐PE, and anti‐F4/80‐PE‐CY7 antibodies were stained at 4 °C for 1 h, repeatedly washed with PBS, and analyzed by flow cytometry.

### Biosafety Evaluation In Vivo

Mice were used to evaluate the safety of HPT and ss‐HPT (*n* = 12 mice per group). Treatment was administered by intraperitoneal injection of HPT and ss‐HPT (20 mg kg^−1^). The animals were observed for 28 days, and half of the mice were sacrificed on the 7th and 28th days. Major organs, including the heart, liver, lung, spleen, and kidney, were harvested, and fixed with 4% paraformaldehyde. Paraffin‐embedded tissue sections were stained with H&E for biosafety evaluation.

### Statistical Analysis

All data were presented as mean ± S. D. Unpaired t‐tests for two groups and ordinary one‐way ANOVA with Tukey's Multiple Comparison test for three or more groups were used to analyze the differences between groups. Differences were considered significant when *p* < 0.05. Associations among variables were determined using Pearson's correlations. Statistical analysis was performed in GraphPad Prism 8.3.

## Conflict of Interest

The authors declare no conflict of interest.

## Supporting information

Supporting Information

## Data Availability

The data that support the findings of this study are available from the corresponding author upon reasonable request.
