# Supplementary material for: Multifunctional Cationic Hyperbranched Polyaminoglycosides that Target Multiple Mediators for Severe Abdominal Trauma Management
Source: Adv Sci (Weinh). 2023 Nov 23;11(1):2305273. doi: 10.1002/advs.202305273 (PMC10767409; doi:10.1002/advs.202305273)
Supplement: Supplementary file 1 — Supporting Information [file ADVS-11-2305273-s001.pdf]

ADVANCED SCIENCE
Open Access

Supporting Information

for *Adv. Sci.*, DOI 10.1002/advs.202305273

Multifunctional Cationic Hyperbranched Polyaminoglycosides that Target Multiple Mediators for Severe Abdominal Trauma Management

Yongqiang Xiao, He Fang, Yuefei Zhu, Jie Zhou, Zhanzhan Dai, Hongxia Wang, Zhaofan Xia, Zhaoxu Tu* and Kam W. Leong**

Supporting Information

Multifunctional Cationic Hyperbranched Polyaminoglycosides that Target Multiple Mediators for Severe Abdominal Trauma Management

Yongqiang Xiao, He Fang, Yuefei Zhu, Jie Zhou, Zhanzhan Dai, Hongxia Wang, Zhaofan Xia, Zhaoxu Tu*, and Kam W. Leong**

Y. Xiao, H. Fang, Z. Dai, Z. Xia
Department of Burn Surgery, the First Affiliated Hospital, Naval Medical University, Shanghai, 200433, China;

Y. Xiao, Y. Zhu, H. Wang, Z. Tu, K. W. Leong
Department of Biomedical Engineering, Columbia University, New York, NY, 10027, USA;

Z. Tu
The Sixth Affiliated Hospital, Sun Yat-sen University, Guangzhou, Guangdong 510655, China

Y. Xiao

ENT institute, Department of Facial Plastic and Reconstructive Surgery, Eye & ENT Hospital, Fudan University, Shanghai, 200031, China.

J. Zhou
Department of Breast Surgery, Affiliated Cancer Hospital and Institute of Guangzhou Medical University, Guangzhou 510095, PR China

K. W. Leong
Department of Systems Biology, Columbia University Medical Center, New York, NY 10032, USA

Table of contents

1. Experimental 3
2. The analysis of cfNAs in trauma patients 10
3. Synthesis of HPT and ssHPT 11
4. Characterizations of HPT and ssHPT 13
5. Degradation of HPT and ssHPT 14
6. DAMPs-induced TLRs activation and cytokines generation 15
7. HPT and ssHPT reduced TLRs activation 16
8. DAMPs-induced TLRs activation and cytokines generation 17
9. HPT and ssHPT reduced CpG-induced cytokines generation 17
10. Cellular uptake of HPT and ssHPT 18
11. HPT and ssHPT reduced plasma clotting in a kaolin-based APTT analysis 19
12. DAMPs-induced plasma clotting 19
13. HPT and ssHPT reduced NETs-induced plasma clotting 20
14. Body weight of mice after treatment 20
15. M2 macrophages analysis in peritoneal fluid of CLP mice 21
16. Blood biochemistry analysis of mice in different treatment groups 21
17. The biocompatibility of HPT and ssHPT in healthy mice 23
18. References 25

1. Experimental

Materials. Gentamicin sulfate, tobramycin, neomycin sulfate, 2-hydroxyethyldisulfide, glycidyl methacrylate (GMA), tetrabutylammonium bromide, ethylene glycol diglycidyl ether (EGDE), ethylenediamine (ED), DL-1,4- dithiothreitol (DTT), triethylamine (TEA, 98%), fluorescein isothiocyanate (FITC), cell counting kit-8 (CCK-8) assay, 4',6-diamidino-2-phenylindole (DAPI), methanol, N,N-dimethylformamide, and, n-hexane were purchased from Fisher Scientific and used without additional purification. Polyamidoamine generation 3.0 (PAMAM-G3) was purchased from Millipore-Sigma (US). Human and Murine IL-6 and TNF-α ELISA Kit were purchased from Invitrogen (US). Cyanine 5-NHS ester (Cy5) was purchased from Lumiprobe Corporation (FL, USA). Milli-Q water was used in all experiments.

Methods. NMR spectra were measured with a Jeol Eclipse (USA) nuclear magnetic resonance spectrometer (500 MHz). UV-vis absorption spectra were recorded on a U-3310 spectrophotometer (Hitachi, Japan). Zeta potential and DLS data were obtained with a Malvern NANO ZSPO in corresponding conditions. AFM was performed using a Bruker Dimension FastScan AFM. Quanti-Blue and CCK8 assays were performed using a microplate reader (Bio-Tek, Winooski, VT). Confocal laser scanning microscopy experiments were conducted with a Nikon Ti Eclipse inverted microscope with A1 scanning confocal unit. The flow cytometer was performed using a BD FACS caliber flow cytometer. *In vivo* and *ex vivo* biodistribution fluorescent images were measured with an IVIS Spectrum system (PerkinElmer, USA). H&E staining slices were imaged using an automated slice scanning system (AxioScan.Z1, Zeiss).

Synthesis of hyperbranched polyaminoglycosides: The detailed synthesis steps of HPT and ssHPT were described in our previous study.[1] To synthesize ssHPT, hydroxyethyl disulfide diglycidyl ether (HDDE) (665 mg, 12.5 mmol) was added to 5 mL of DMSO containing tobramycin (467 mg, 1mmol) to reach an equal stoichiometric ratio of amine and epoxy

groups. The reaction mixture was degassed for 10 min by bubbling a stream of nitrogen gas, then 300 mL of TEA was added. Next, the reaction was carried out at 40 °C with constant stirring under the protection of a nitrogen atmosphere. After 24 h, an excess amount of ED (1.0 mL) was added to the mixture to terminate the hyperbranching reaction by reacting for another 1 h at 60 °C. The crude products were dialyzed (MWCO 1000) against deionized water to remove unreacted small molecules, and the tobramycin-based hyperbranched polyaminoglycosides (ssHPT) were obtained after lyophilization. Similarly, the disulfide-free tobramycin-based polyaminoglycosides (HPT) were synthesized by using EGDE (instead of HDDE) as a diglycidyl linker.

DNA binding efficiency assay: The DNA binding ability of HPT and ssHPT was determined by Picogreen competitive binding experiment.[2] Picogreen is a fluorescent dye for double-stranded DNA (dsDNA) determination, and its fluorescence intensity will be significantly reduced when DNA is competitively bound with nanomaterials. In brief, the diluted Picogreen reagent and DNA in 1×TE buffer were first mixed and incubated for 10 minutes in the dark at room temperature. Then the different concentrations of HPT or ssHPT were added to the mixture. After shaking and incubation for 30 minutes at room temperature in the dark, fluorescence intensity was detected using a Multiwall Plate Reader. The binding efficiency of the cationic material to DNA was calculated.

Biodegradation tests: ssHPT was labeled with FITC and dispersed in PBS (4 mL, pH 7.4), and then it was equally transferred into three dialysis tubes (spectra/Por MWCO 1 kD). Afterwards, the tubes were immersed in two vials (3 vials contained 30 mL PBS without GSH, 30 mL PBS with 1 mM GSH and 30 mL PBS with 10 mM GSH, respectively). Vials were setup in a thermostatic water bath (37 °C) on a heating rotator. Samples (0.1 mL) from vial medium were used for fluorescence measurements after 0 h, 3 h, 6 h, 9 h, 12 h and 24 h incubation. The amount of degraded polymer at different time frames was determined by a microplate reader, with excitation at 480 nm and emission at 550 nm and using the calibration

curve. The degradation studies were performed in triplicate. The degradation profiles of HPT were measured in the same way.

Cell culture: Human dermal fibroblast and RAW 264.7 cells (purchased from ATCC) were maintained in DMEM Medium supplemented with 10% FBS, and 1% Penicillin-Streptomycin. HEK-blue™ hTLR3, HEK-blue™ hTLR8 and HEK- blue™ hTLR9 reporter cell lines were purchased from InvivoGen, San Diego, CA, and were maintained by following the manufacturer's protocol. THP-1 cells (purchased from ATCC) were maintained in RPMI 1640 medium supplemented with 10% (v/v) FCS, 2 mmol/l glutamine, and 1% Penicillin-Streptomycin. All these cells were incubated at 37℃ in a humidified atmosphere with 5% CO_2.

Cytotoxicity test: the cytotoxicity of the HPT and ssHPT was evaluated using the Cell Counting Kit-8 assay (Dojindo Molecular Technologies, Inc., Rockville, MD, USA). The normal 5×10^3 HDFs were seeded in a 96-well plate and cultured at 37 °C in a humidified atmosphere with 5% CO_2. When the cell density reached 70-80%, the medium was replaced by a medium with different concentrations of polycationic materials. After 24 h incubation, the medium was replaced again with a medium containing 10% CCK-8 reagent and incubated for 2-3 h in a 37°C incubator under 5% CO_2. Then the absorbance of the solution was read at 450 nm using a Multiplate Reader (BioTek Instruments, Winooski, VT, USA).

HDF DAMPs: HDF DAMPs were generated by the freezing-thawing method based on the previous report.[3] In brief, 1×10^6 HDF cells were harvested and washed with PBS three times, then 200 μL of molecular grade water was added and vortexed for 10 seconds at maximum speed, then frozen in -80℃ for 24h and thawing at room temperature and then vortexed 10 seconds each interval, totally for 5 cycles. The levels of cfDNA inside the DAMPs were determined using the Quant-iT PicoGreen DNA assay kit (Thermo Fisher, Waltham, MA) following manufacturer's introduction. The genomic DNA and total RNA

were isolated from the HDF DAMPs using AllPrep DNA/RNA Micro Kit (Qiagen, Valencia, CA). The concentration and quality of isolated DNA and RNA were measured by a Nanodrop machine.

Inhibition of the activation of TLRs by extracellular agonists: The inhibitory efficiency of cfNA-mediated TLRs activation by HPT and ssHPT was investigated using the HEK Blue^{TM} reporter cells, which are originated and constructed by co-transfection of HEK 293 cells with hTLRs gene and optimized embryo-secreted alkaline phosphatase (SEAP) reporter gene.[2] The SEAP activity was assessed using the alkaline phosphatase detection reagent, QUANTI-Blue™, by detecting OD at 620 nm. Poly (I:C) (LMW), ORN 06 and CpG-ODN were used respectively as synthetic analogs of double strain RNA (dsRNA), single strain RNA (ssRNA) and non-methylated DNA, and acted as TLR3, TLR8 and TLR 9 agonists respectively to mimic the cfNAs-induced inflammation.

First, to study the inhibition of nucleic acid-sensing TLRs (TLR3 and 9) activations by cationic materials in HEK Blue^{TM} reporter cells, different numbers of cells were seeded into 96-well plates, respectively, 5×10^4 for TLR 3, 4×10^4 and 8×10^4 for TLR 9 each well. 6-8 h later, after the cells were attached to the wall, then 1μg/mL of Poly (I:C) (LMW), ORN 06 or CpG ODN 2006 and different cationic materials were respectively added to the plate. After incubation for 24 h, 50 μL supernatants of each well were collected and incubated together with 150μL QUANTI-Blue^{TM} medium for 1-6 h in a new transparent 96 well plate. Then the absorbance was measured at 620 nm using a Multiwall Plate Reader (BioTek Instruments, Winooski, VT, USA). The relative TLR activation was calculated by (X)/(X0), where X was the OD value of the experimental group, and X0 was the control group.

Inhibition of the activation of TLRs by HDF DAMPs: TLR reporter cells were incubated with 1μg/mL (cfDNA concentration) HDF DAMPs with or without polycationic material in a 96-well plate. After 24h incubation, 50 μL culture supernatants were harvested and incubated for 3-5 h with 150 μL QUANTI- Blue^{TM} solution in a transparent flat-bottom 96-well plate.

Then the absorbance was measured at 620 nm using a Multiwall Plate Reader (BioTek Instruments, Winooski, VT, USA). The relative TLR activation was calculated.

Flow cytometry: the inhibition of CpG DNA endocytosis by cationic material was detected in murine macrophages (RAW264.7). In brief, after the cells were seeded on a 24-well plate and the cell grew 70-80%, 1 μg/mL FITC-labeled CpG DNA, and 5 μg/mL cationic material were added. After incubation for 24 h, cells were harvested, and the fluorescence intensity of FITC-CpG was detected using flow cytometry (MoFlo XDP, Beckman, USA).

Cellular colocalization: 2×10^4 RAW 264.7 cells per well were seeded on the confocal slide inside a 24-well plate. 24h later, 1 μg/mL FITC-labeled CpG, and 5 μg/mL Cy5-labeled polycationic material were added. After incubation for 24 h, the supernatant was discarded, and cells were washed with PBS three times. Then medium containing LysoTracker™ Red (Thermo Fish, USA) was added and then incubated for 20 minutes. After washing three times with PBS, cells were fixed using 4% paraformaldehyde at room temperature for 30 minutes. The nucleus was then dyed with 1×DAPI at room temperature for 20 minutes, away from light. Then the film was sealed with neutral gum. The image was taken using a Confocal laser scanning microscopy (Leica, DMI3000B, Germany).

HDF Mitochondria DAMPs (MTD) and mtDNA: First, mitochondria were isolated from human dermal fibroblasts using the Mitochondrial Isolation Kit for Cultured Cells (Thermo Scientific, West Palm Beach, FL, USA) by following the manufacturer's protocol. The isolated mitochondrial pellet was dissolved using molecular grade water. The freezing-thawing method was also performed to generate the mitochondrial DAMPs, just like the HDF DAMPs production. The DNA concentration of MTD was detected using the Quant-iT™ PicoGreen™ dsDNA Kit, following the manufacturer's protocol. The mitochondrial DNA was isolated from MTD using DNeasy Blood & Tissue kit (Qiagen, Valencia, CA) following the manufacturer's protocol. The concentration and the quality of isolated mtDNA were detected by using a Nanodrop machine.

ELISA assay: First, to study the polycationic material inhibition of immune stimulatory effects caused by CpG DNA, RAW264.7 cells were used, 2×10^5 RAW 264.7 cells per well were added to a 24-well plate. After cell attachment, 1 μg/mL of CpG DNA together with different concentrations of cationic materials were added. Second, to study the inhibition effect of cationic materials on mtDNA-induced inflammation, THP-1 cells were used and stimulated by 40 ng/mL Propylene glycol monomethyl ether acetate (PMA) for 48h to induce differentiation into human macrophages. Then different concentrations of HDF DAMPs, DAMPs derived RNA or mtDNA with or without cationic materials were added to the 24-well plate. The supernatants were collected after 24 h incubation, and the TNF-α, IL-1β and IL-6 concentrations were detected using ELISA kits.

Antibacterial activity in vitro: (1) Contact sterilization experiments: 20 μL HPT or ssHPT with different concentrations were put into the 5 mL Eppendorf tube, and then *E. coli* or *S. aureus* suspension (3 mL) was added, and incubated at 37 ℃, shaking (220 rpm/min). 100 μL of bacterial culture medium was collected at 0, 2, 4, 8, 12, 16, and 24 h, and the OD value at 600 nm was measured. The bacterial growth curve was then plotted. (2) Surface antibacterial activity: Autoclaved nutrient agar (10 mL) was added to 60-mm Petri dishes to form a solid culture medium. Bacterial solutions of *E. coli* and *S. aureus* (20 μL) together with 20 μL HPT or ssHPT with different concentrations were uniformly dispersed on the medium surface. After incubation (37 °C, 24 h), images of the plates were recorded. Sterile and bacteria-contaminated mediums without polymer were used as negative and positive controls, respectively.

Isolation and identification of neutrophils and induction of NETs: Neutrophils were firstly isolated from healthy human whole blood (Human Whole Blood K2EDTA Pooled Gender, BIOIVT, Hicksville, New York, USA) using MACSxpress Whole Blood Neutrophil Isolation Kit (Miltenyi Biotec, Cologne, Germany) following the instructions and identified them by

their surface markers CD15 (Miltenyi Biotec, Cologne, Germany) and CD16 (Miltenyi Biotec, Cologne, Germany) using flow cytometry. Neutrophils were cultured using Neutrophil Medium W/Kit (Cell Biologics, Chicago, USA) and then induced to form NETs using 50 nM Phorbol-12-myristate-13-acetate (PMA) (Sigma-Aldrich, St. Louis, MO, US) or 10 μg/mL lipopolysaccharide (LPS) (Sigma-Aldrich, St. Louis, MO, US), 10 μg/mL (High Mobility Group Protein 1) HMGB1 or HDF DAMPs treated for 4 h. Then NETs were identified through their specific immunofluorescence staining for Histone H3(Sigma-Aldrich, St. Louis, MO, USA) and DAPI. The image was taken using a Confocal laser scanning microscopy (Leica, DMI3000B, Germany). Then NETs induced human plasma coagulation assays were followed as the above procedure.

Sonoclot evaluation: human Whole blood was collected from healthy volunteers in Changhai Hospital using a 3.2% sodium citrate coagulation test tube. 20 μL HDF DAMPs with or without HPT or ssHPT were added to 360 μL whole blood, followed by the addition of 20 μL $CaCl_2$ (0.25 M), then the complex was immediately placed into disposable Sonoclot cuvette to initiate Sonoclot coagulation analysis.

Statistical analysis: Data were expressed as mean ± s. d.. Differences between groups were analyzed using Student's t-test or one-way analysis of variance when comparing only two groups or more than two groups. $P < 0.05$ was considered representative of a statistically significant difference. GraphPad Prism 8 was used to perform the statistical analysis.

2. The analysis of cfNAs in trauma patients

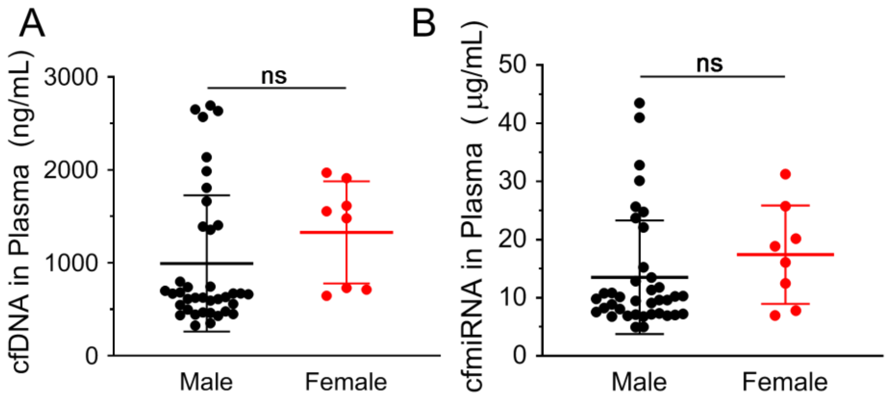

Figure S1. The levels of cfDNA (A) and cfmiRNA (B) in male (n=38) and female sepsis patients (n=8). (ns, no significance)

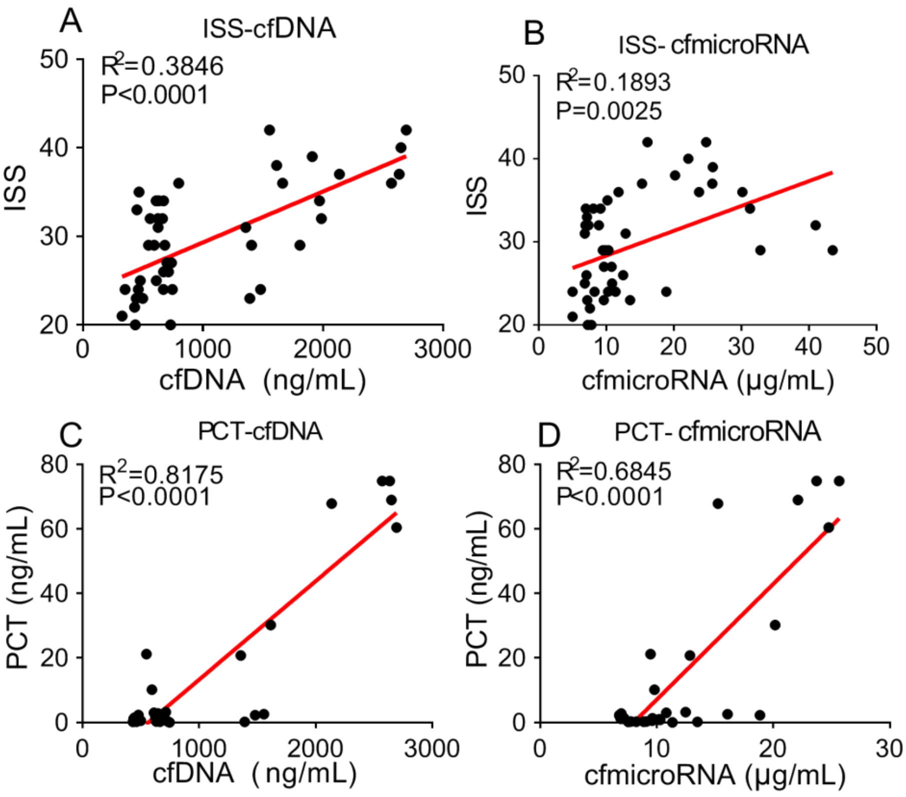

Figure S2. (A) The correction between cfDNA level and Injury Severity Score (ISS) of trauma patients (n = 46). (B) The correction between cfmiRNA level and Injury Severity Score (ISS) of trauma patients(n = 46). (C, D) The correction between cfDNA (C), cfmiRNA (D) level, and PCT concentrations in plasma from trauma patients (n = 34).

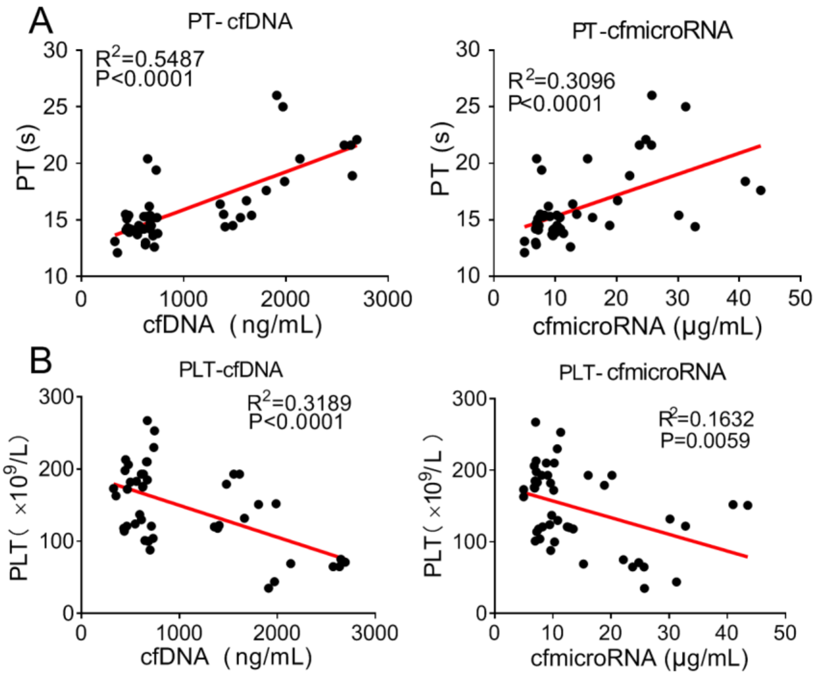

Figure S3. (A) The correction between cfDNA, cfmiRNA level and PT of the plasma from trauma patients. (n = 45) (B) The correction between cfDNA, cfmiRNA level, and PLT in blood from trauma patients. (n = 45)

3. Synthesis of HPT and ssHPT

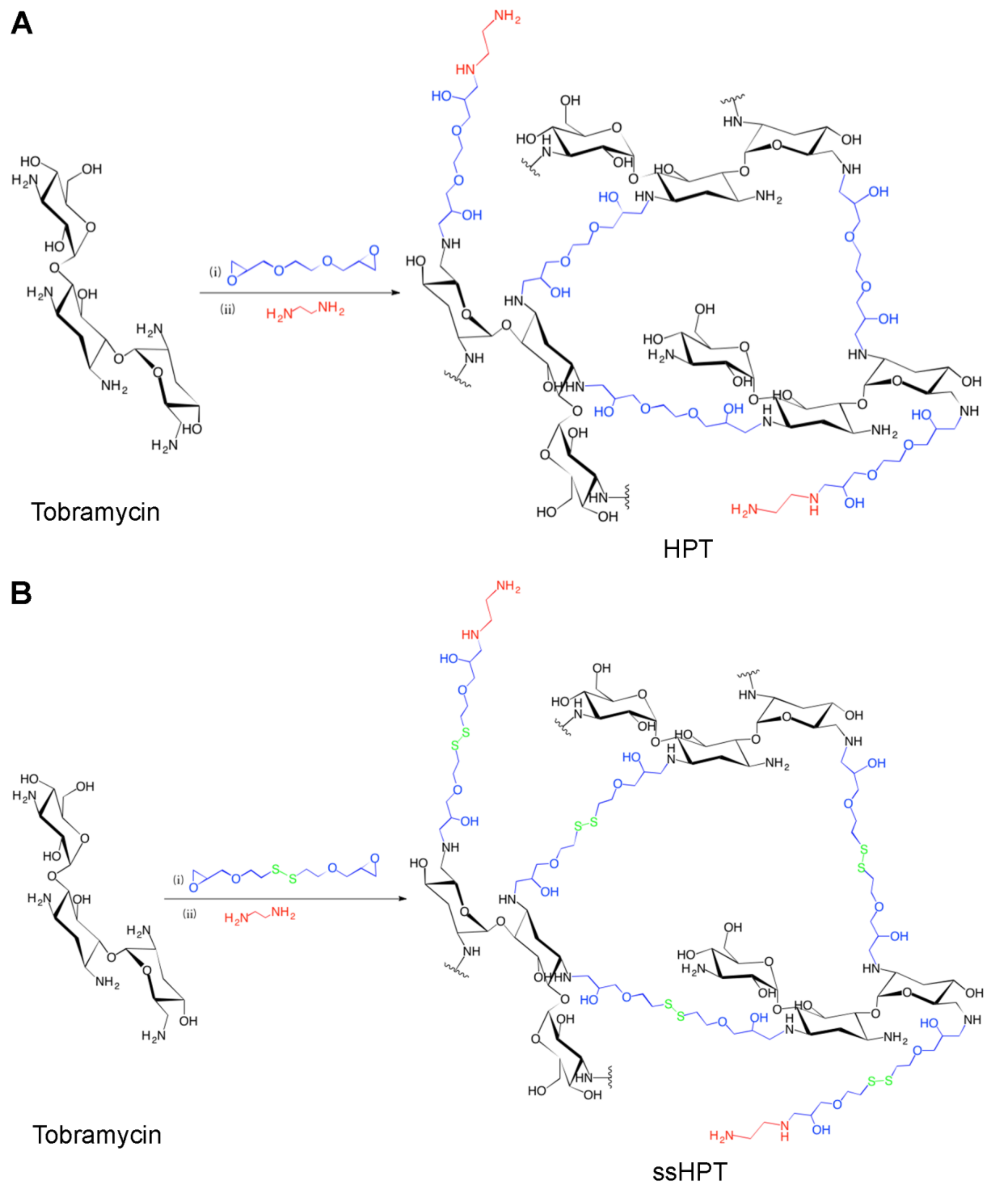

Scheme S1. Synthesis routes for (A) HPT and (B) ssHPT. Detailed methods are provided in the Experimental section above.

4. Characterizations of HPT and ssHPT

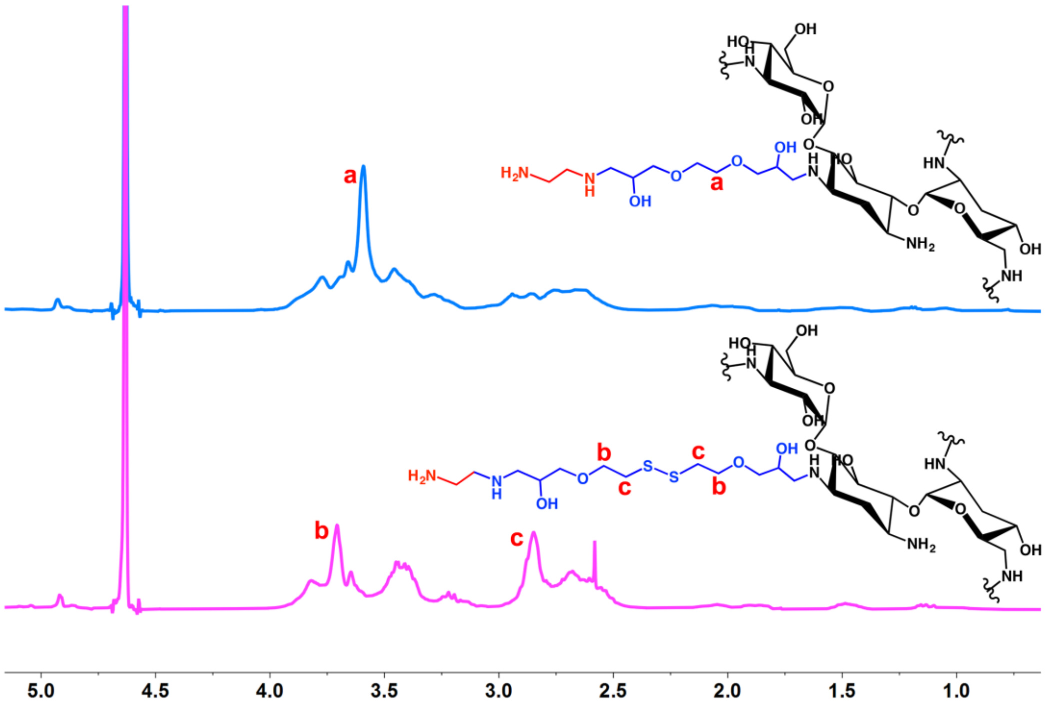

Figure S4. ^{1}H NMR data of HPT and ssHPT in D_2O. Signals at 4.7 ppm are attributed to the protons of D_2O.

5. Degradation of HPT and ssHPT

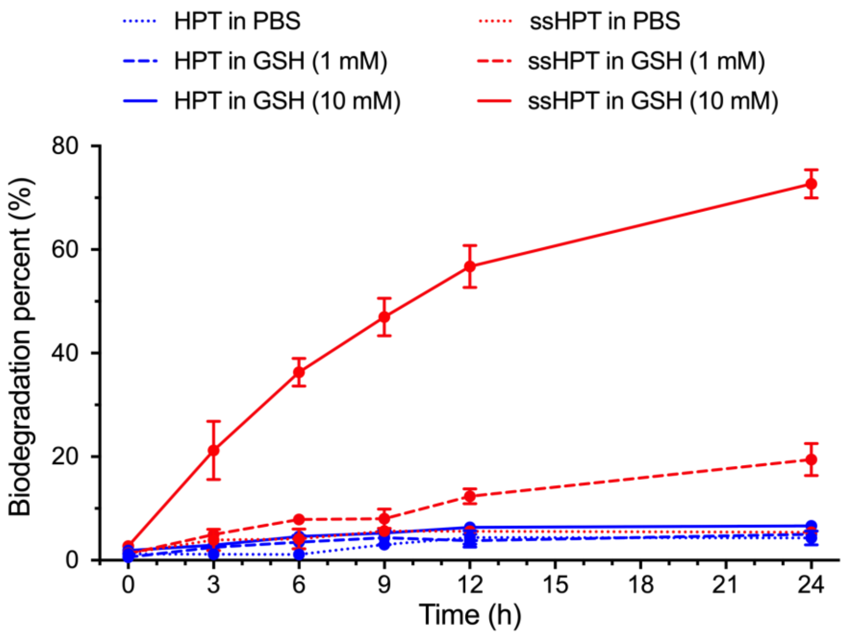

Figure S5. Biodegradation of HPT and ssHPT in PBS (7.4) at 37 ℃ within 24h (n = 3) .

6. DAMPs-induced TLRs activation and cytokines generation

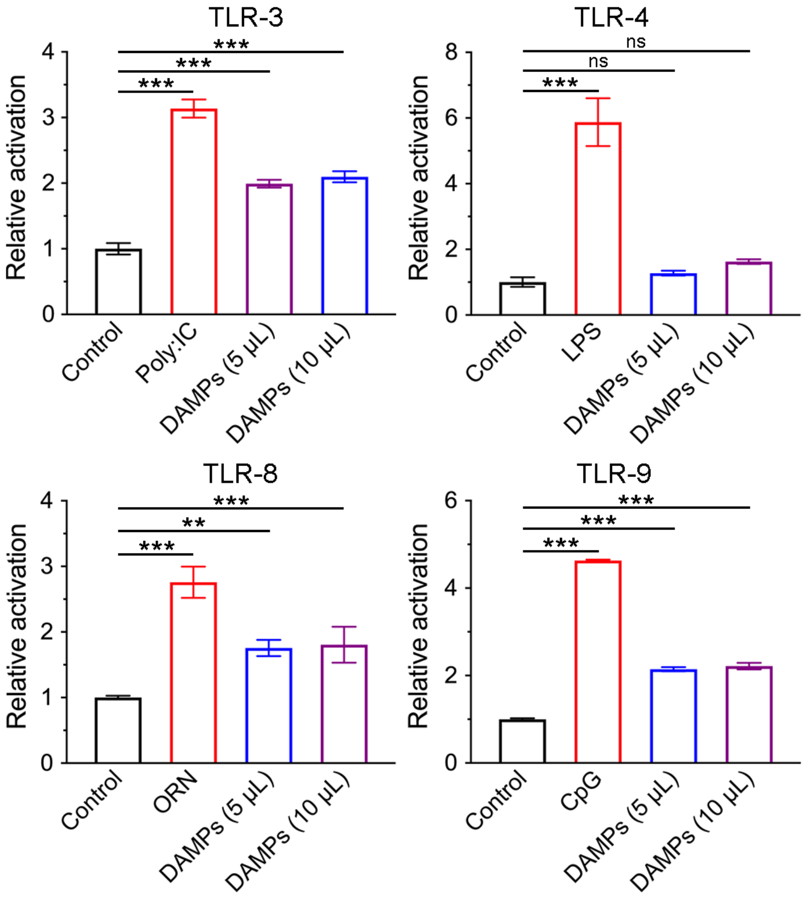

Figure S6. DAMPs-induced TLR-3, TLR-4, TLR-8 and TLR-9 activation in HEK-TLR cells. Poly (I:C), LPS, ORN and CpG were applied as the positive control. Data represent mean ± s. d. (n = 3) (**p*<0.05, ***p*<0.01, ****p*<0.001).

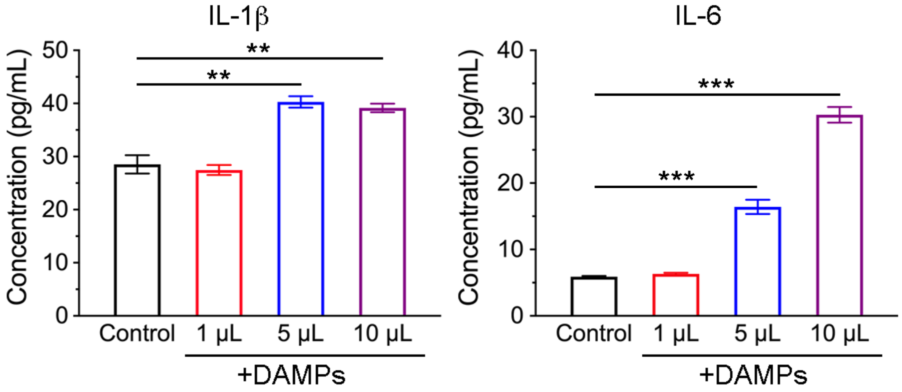

Figure S7. DAMPs-induced IL-1β and IL-6 generation in THP-1 derived macrophage. Data represent mean ± s. d. (n = 3) (*p<0.05, **p<0.01, ***p<0.001).

7. HPT and ssHPT reduced TLRs activation

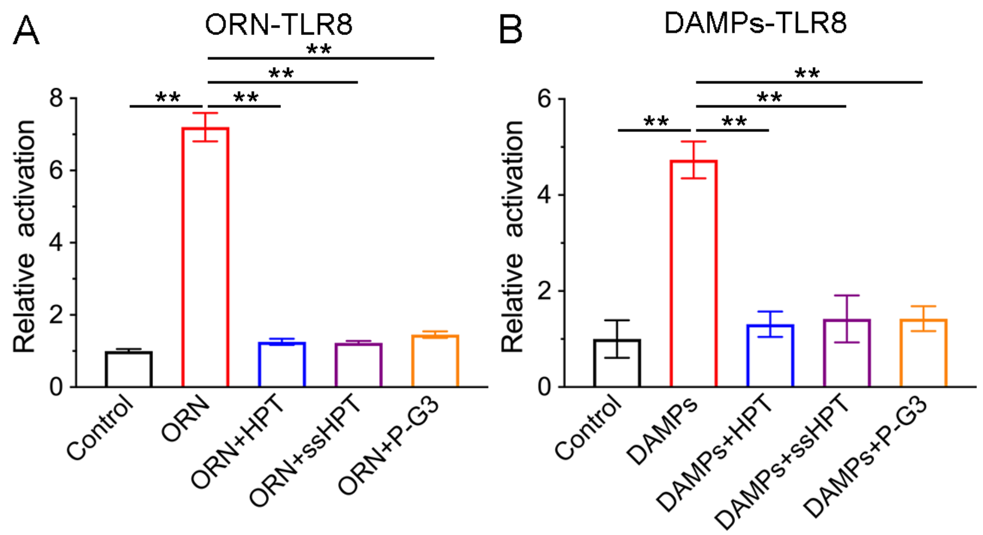

Figure S8. HPT, ssHPT, and P-G3 reduced (A) ORN-induced TLR8 activation and (B) DAMPs-induced TLR-8 activation in HEK-TLR cells. Data represent mean ± s. d. (n = 3) (*p<0.05, **p<0.01, ***p<0.001).

8. DAMPs-induced TLRs activation and cytokines generation

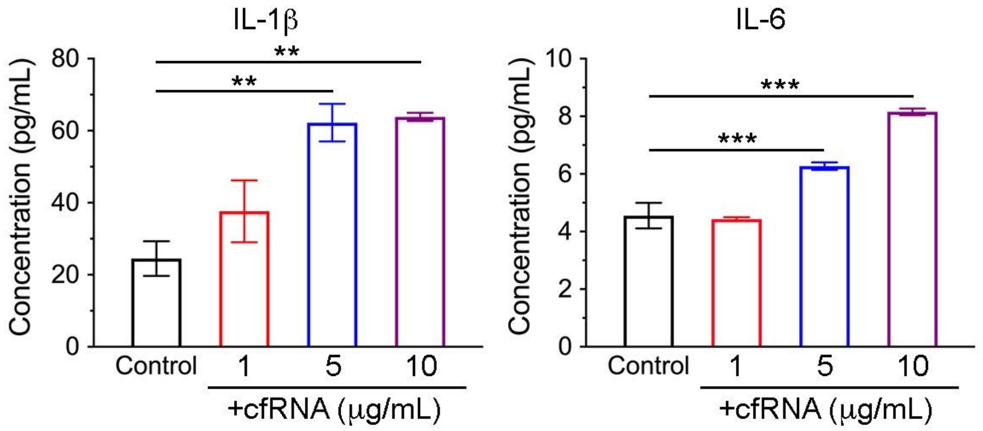

Figure S9. cfRNA-induced IL-1β and IL-6 generation in THP1 derived macrophage. cfRNA was isolated from DMAPs by cfRNA isolation kit. Data represent mean ± s. d. (n = 3) (**p*<0.05, ***p*<0.01, ****p*<0.001).

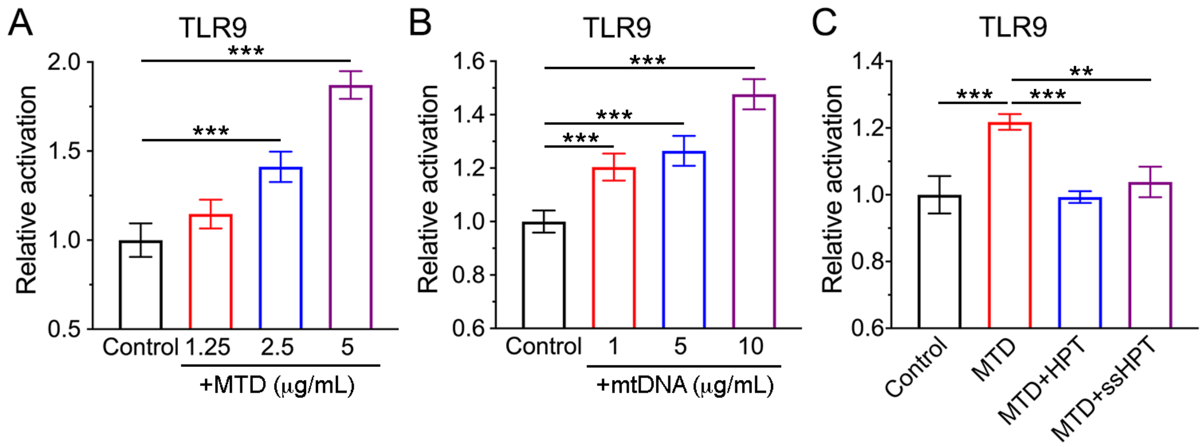

Figure S10. (A) Mitochondria DAMPs (MTD)-induced TLR-9 activation in HEK-TLR cells. (B) Mitochondria DNA (mtDNA)-induced TLR-9 activation in HEK-TLR cells. (C) HPT and ssHPT reduced MTD-induced TLR9 activation in HEK-TLR cells. Data represent mean ± s. d. (n = 3) (**p*<0.05, ***p*<0.01, ****p*<0.001).

9. HPT and ssHPT reduced CpG-induced cytokines generation

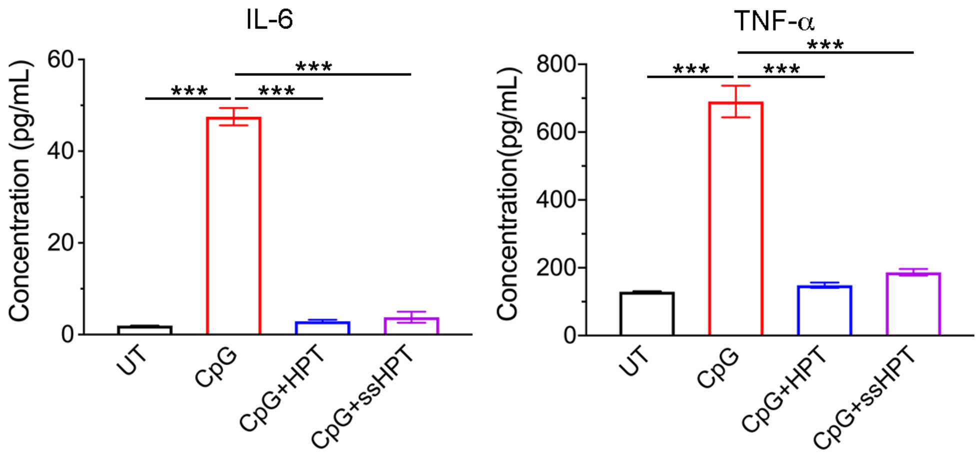

Figure S11. HPT and ssHPT reduced CpG-induced IL-6 and TNF-α generation in RAW264.7 cells. Data represent mean ± s. d. (n = 3) (*p<0.05, **p<0.01, ***p<0.001).

10. Cellular uptake of HPT and ssHPT

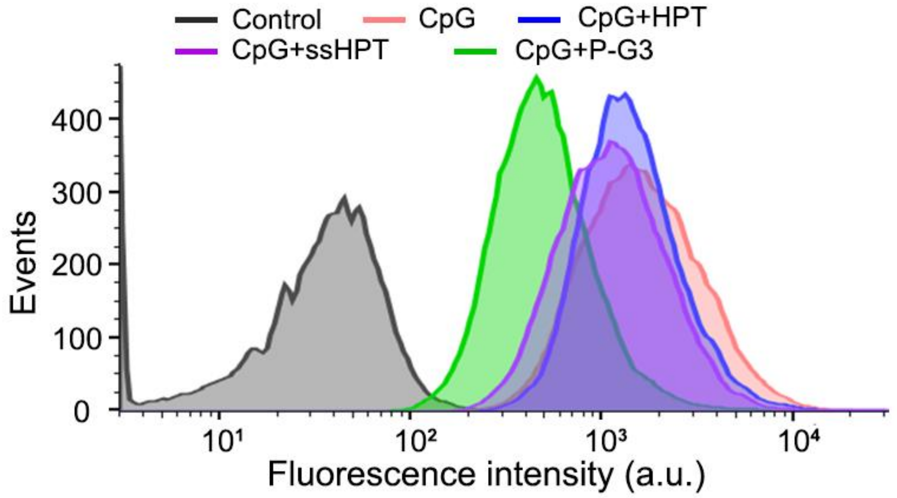

Figure S12. Flow cytometry of RAW264.7 cells after 24 h incubation with CpG, CpG+HPT, CpG+ssHPT, or CpG+P-G3. CpG was labeled with FITC. RAW264.7 cells without treatment were used as a control.

11. HPT and ssHPT reduced plasma clotting in a kaolin-based APTT analysis

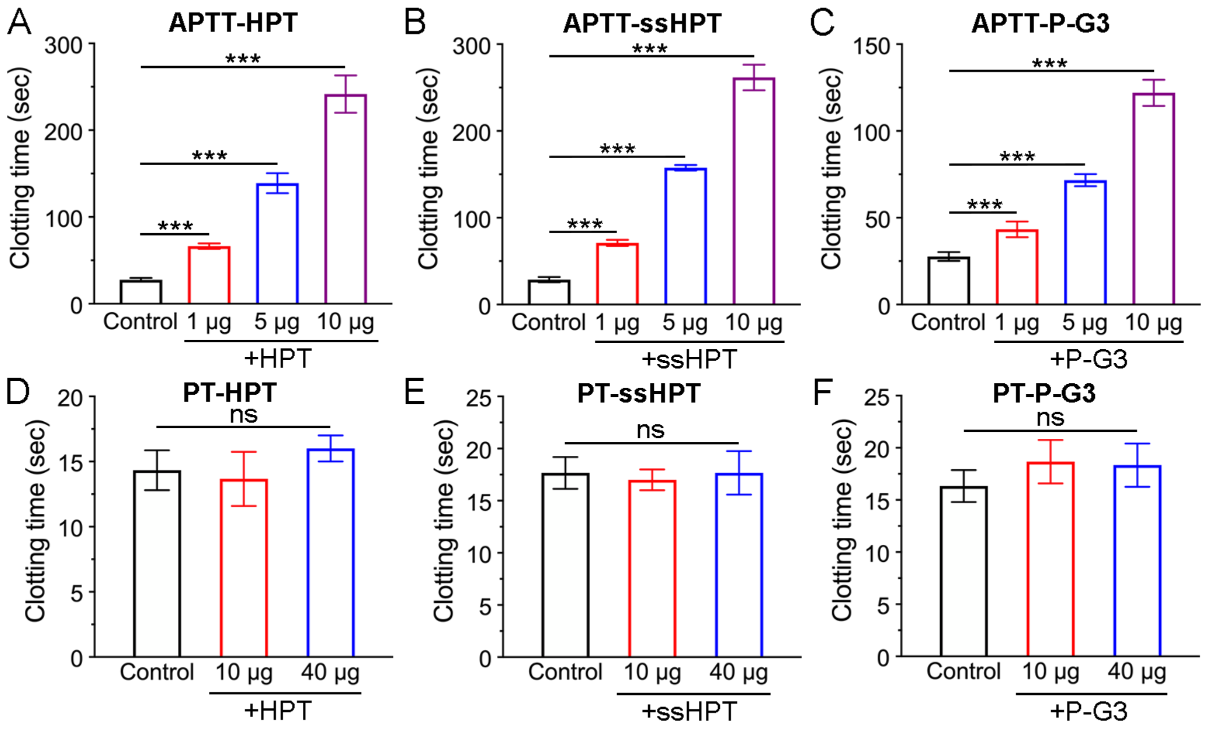

Figure S13. The APTT clotting test of human plasma after incubation with (A) HPT, (B) ssHPT and (C) P-G3. The PT clotting test of human plasma after incubation with (D) HPT, (E) ssHPT and (F) P-G3. Data represent mean ± s. d. (n = 3) (**p*<0.05, ***p*<0.01, ****p*<0.001).

12. DAMPs-induced plasma clotting

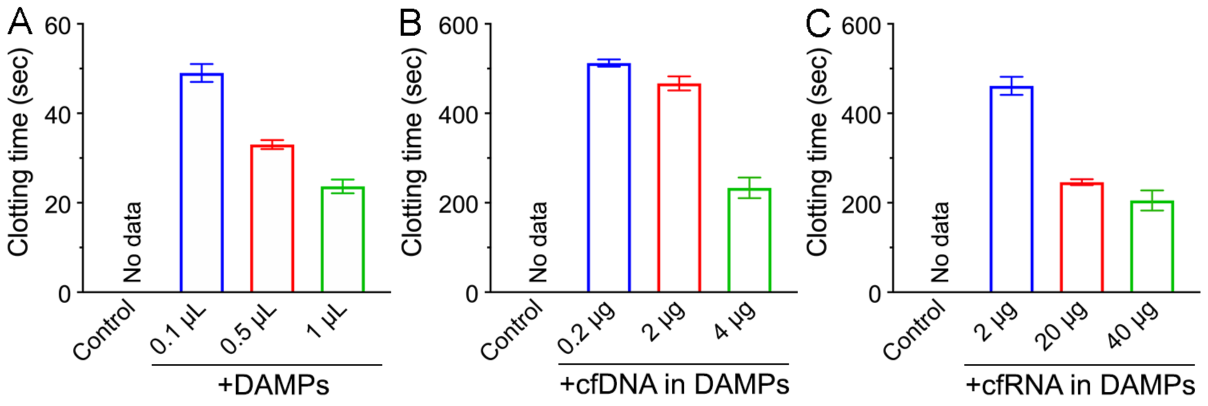

Figure S14. The clotting time of normal pooled human plasma after incubation with (A) DAMPs, (B) cfDNA in DAMPs and (C) cfRNA in DAMPs. (n = 3)

13. HPT and ssHPT reduced NETs-induced plasma clotting

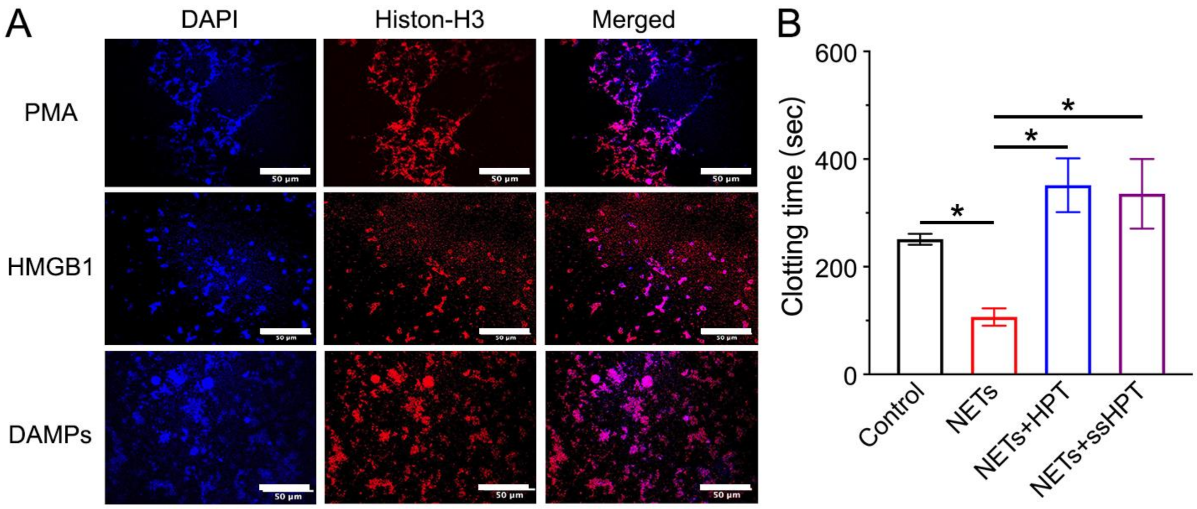

Figure S15. (A) Representative photographs with allocated fluorescent colors representing DAPI and CitH3 in human neutrophils treated with PMA, HMGB1, and DAMPs. Scale bar: 50 μm. (B) The clotting time of human plasma after incubation with NETs, NETs+HPT, and NETs+ssHPT. Data represent mean ± s. d. (n = 3) (*p<0.05, **p<0.01, ***p<0.001).

14. Body weight of mice after treatment

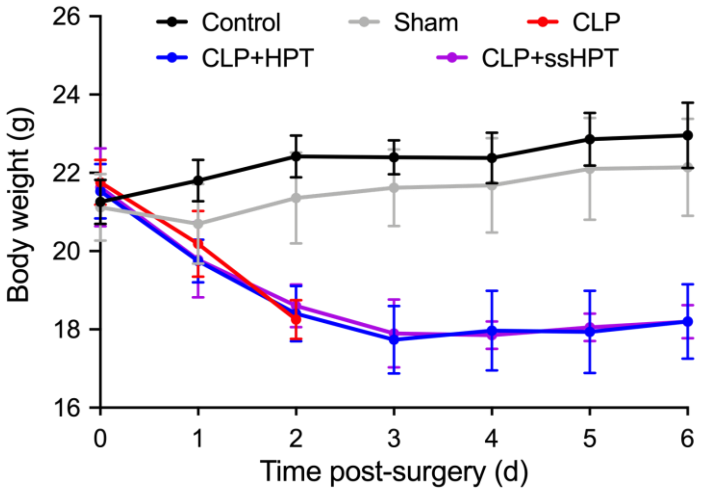

Figure S16. Body weight of mice in different treatment groups recorded for 6 d after CLP. (n = 10)

15. M2 macrophages analysis in peritoneal fluid of CLP mice

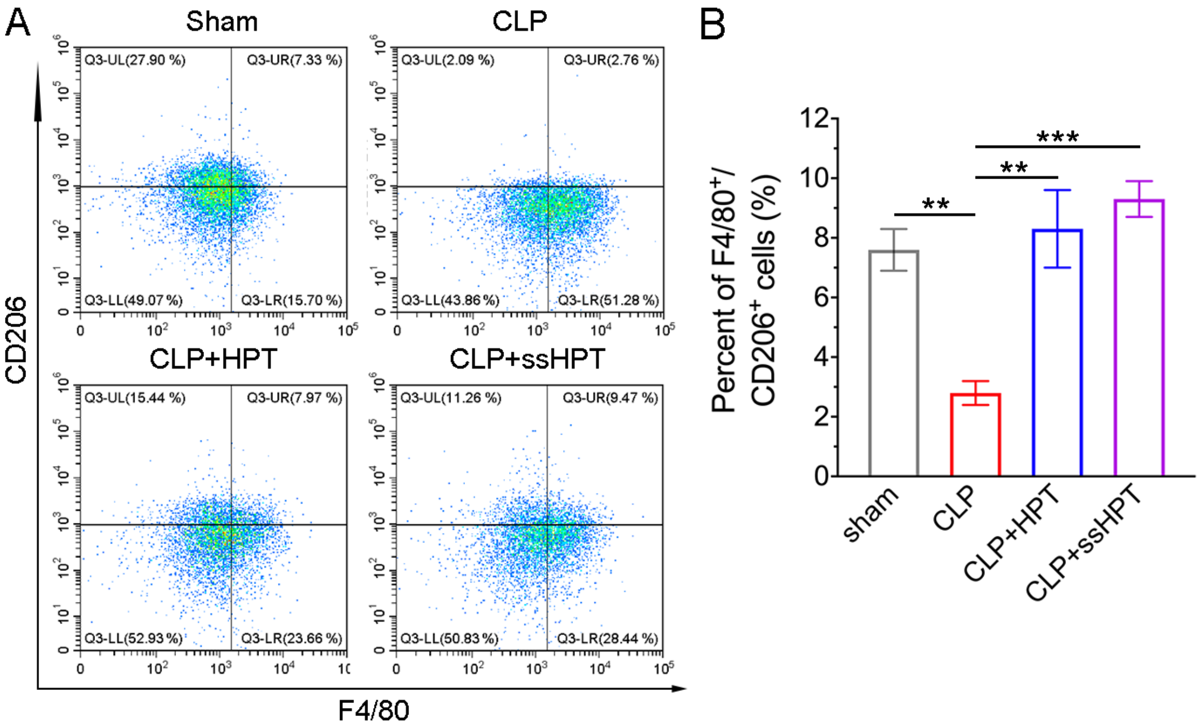

Figure S17. (A) Flow cytometry and (B) quantitative analysis of M2 macrophages in peritoneal fluid of CLP mice in different treatment groups. Data represent mean ± s. d. (n = 3) (**p*<0.05, ***p*<0.01, ****p*<0.001).

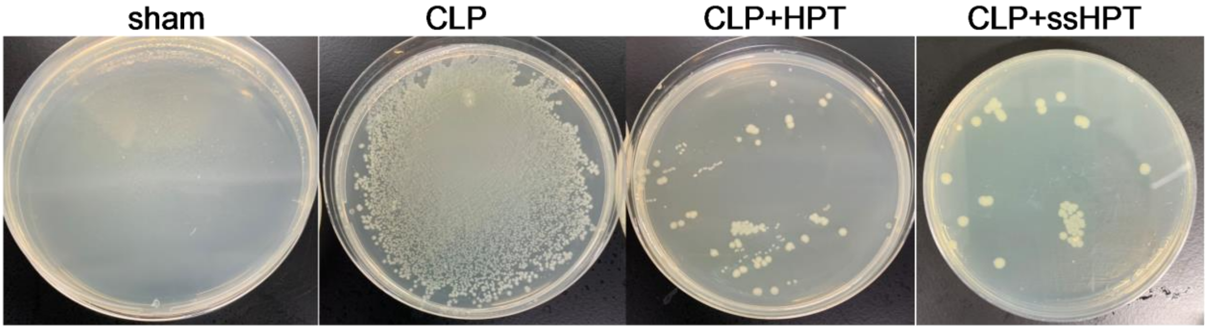

Figure S18. Bacterial colony image from peritoneal cavity of mice in different treatment groups.

16. Blood biochemistry analysis and Inflammatory cytokine infiltration in the lungs of mice in different treatment groups

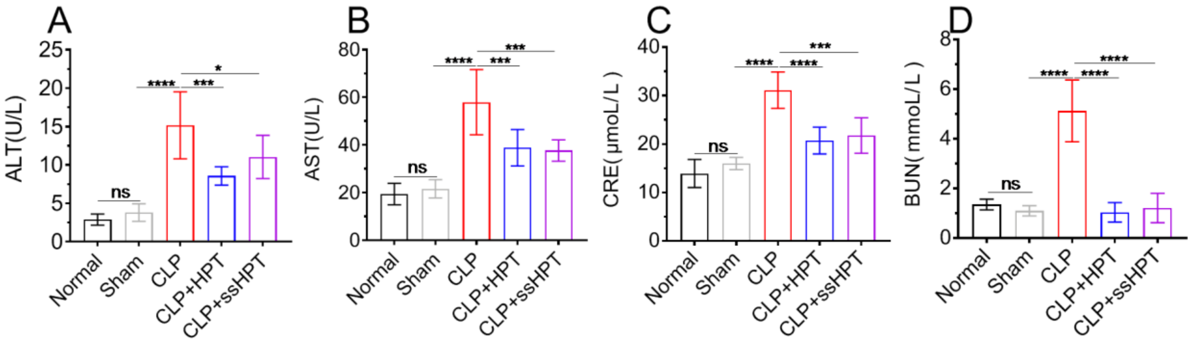

Figure S19. (A) ALT, (B) AST, (C) CRE and (D) BUN in mice serum in different groups. Data represent mean ± s. d. (n = 6) (*p<0.05, **p<0.01, ***p<0.001).

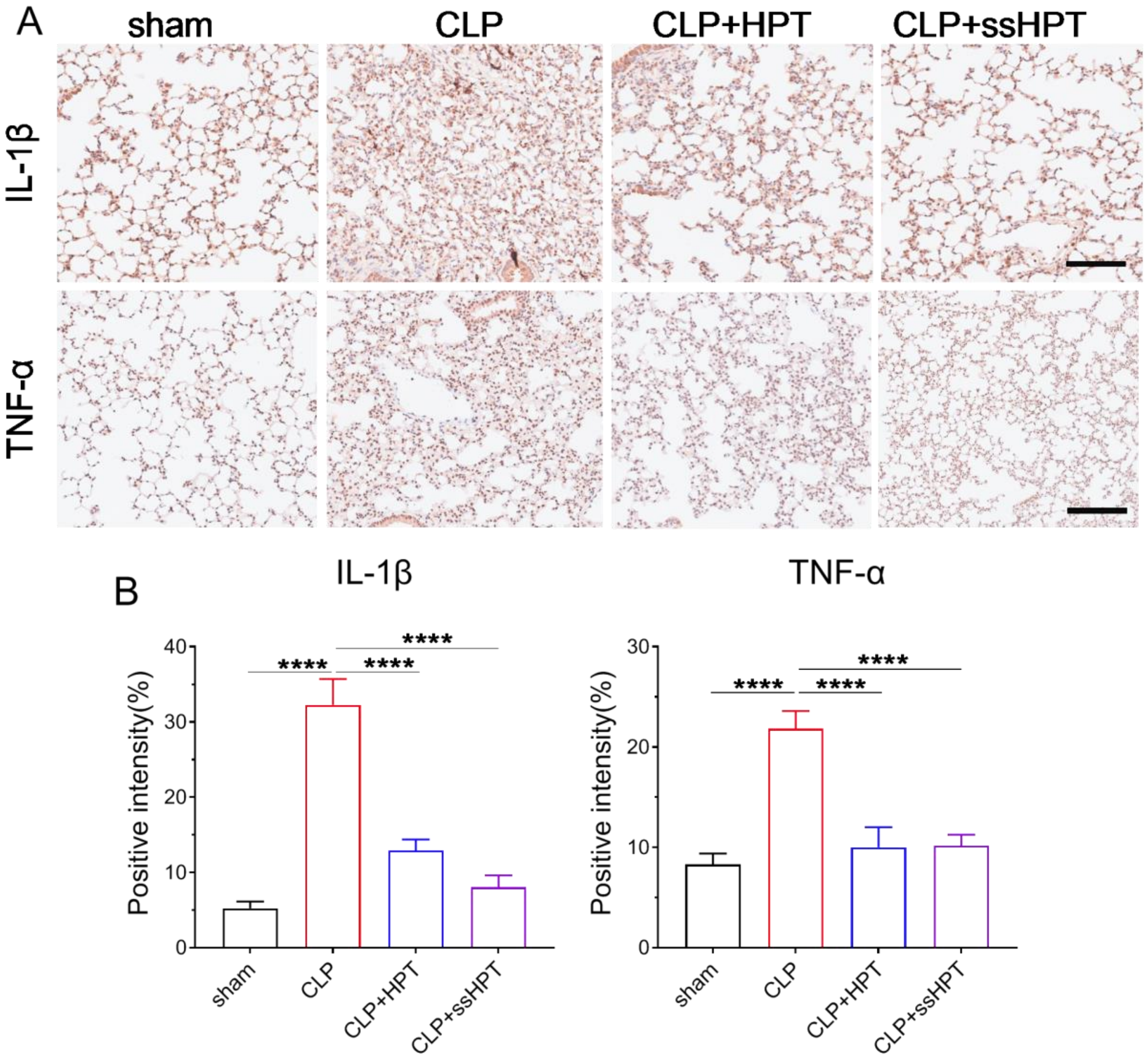

Figure S20. (A) Immunohistochemical staining of TNF-α and IL-1β in the lungs of different treatment groups. Data represent mean ± s. d. (n = 3) (**p*<0.05, ***p*<0.01, ****p*<0.001). Scale bars, 100 μm.

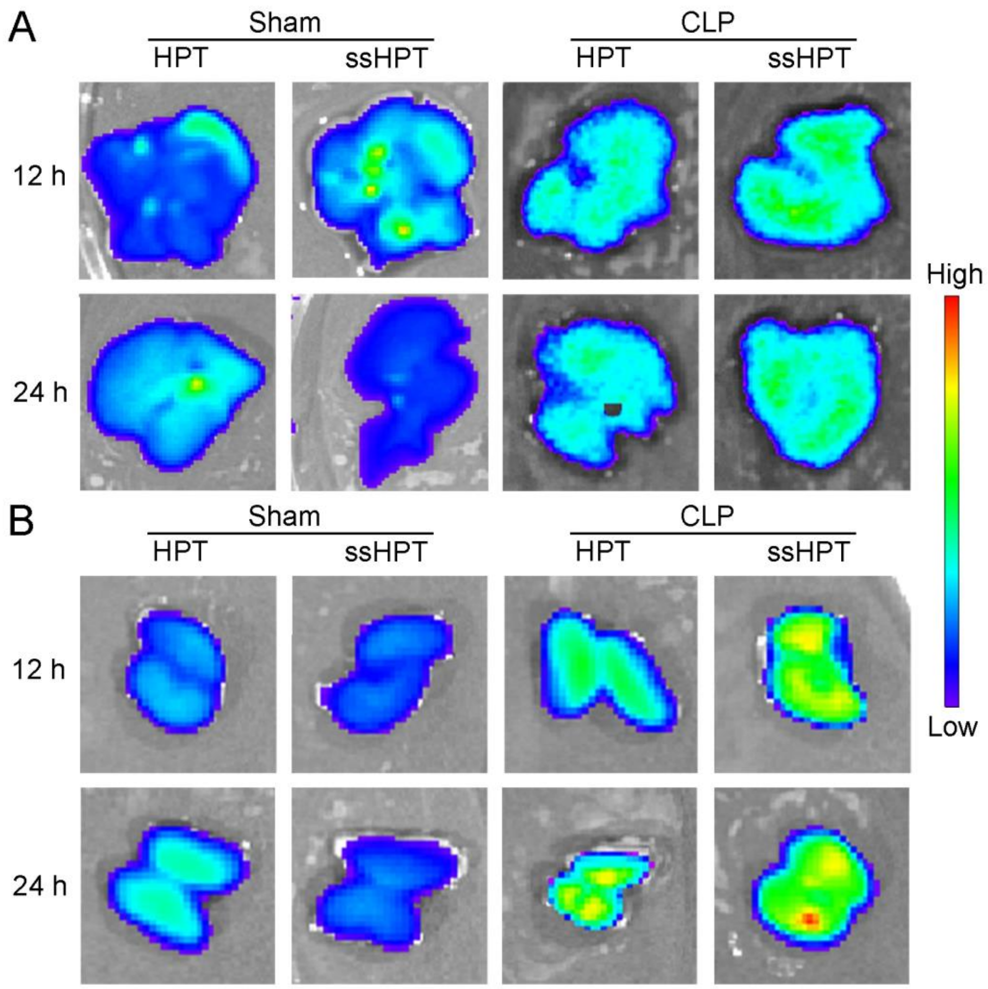

Figure S21. Biodistribution and biodegradation of HPT and ss-HPT in sham mice and CLP mice. *Ex vivo* fluorescence images of (A) livers and (B) kidneys of sham and CLP mice after 12 h and 24 h treatment with HPT and ss-HPT. (n = 3)

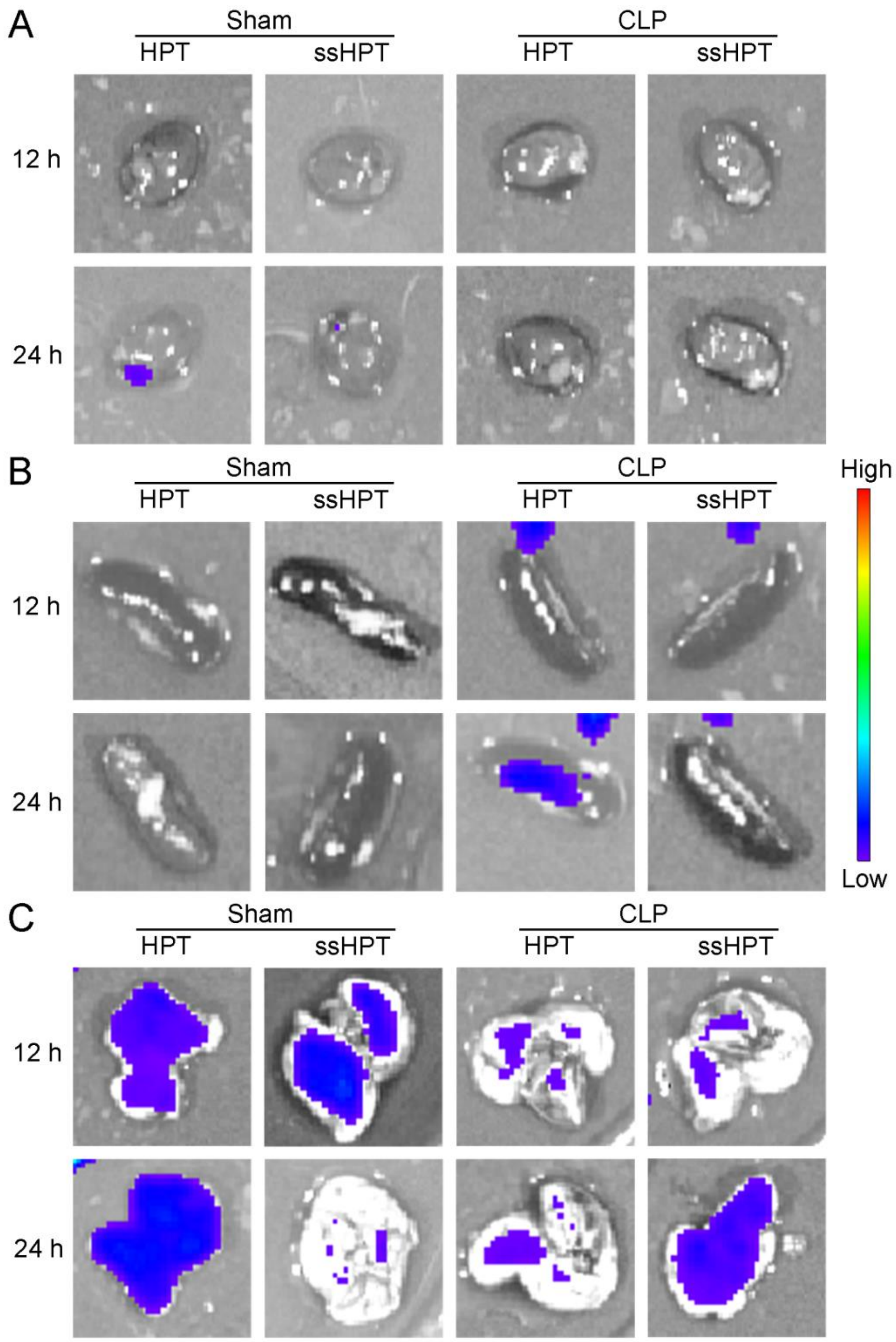

Figure S22. Biodistribution and biodegradation of HPT and ss-HPT in sham mice and CLP mice. *Ex vivo* fluorescence images of (A) hearts, (B) spleens and (C) lungs of sham and CLP mice after 12 h and 24 h treatment with HPT and ss-HPT. (n = 3)

17. The biocompatibility of HPT and ssHPT in healthy mice

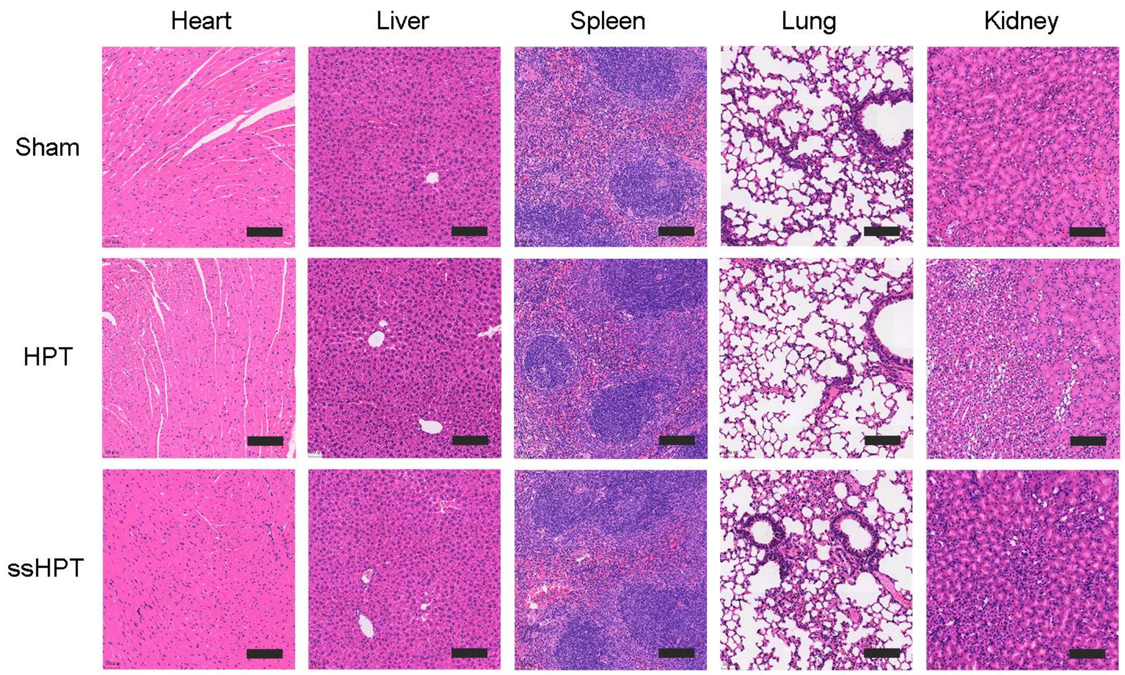

Figure S23. H&E staining of hearts, livers, spleens, lungs, and kidneys from healthy mice after different treatments for 7 days. Scale bars, 200 μm.

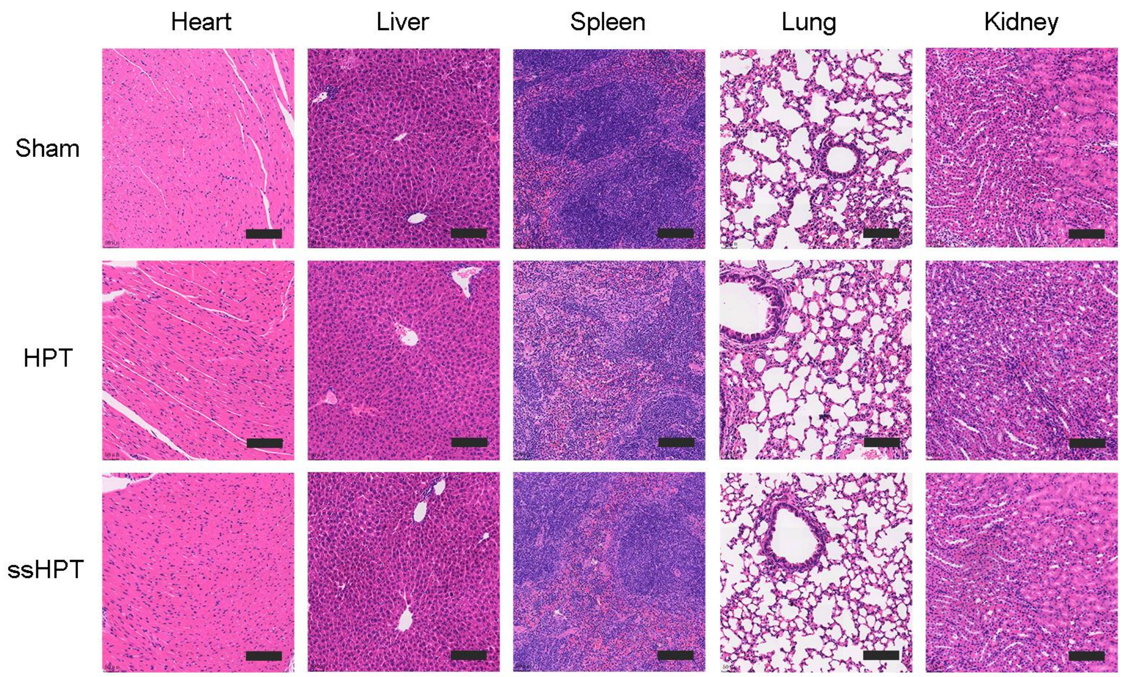

Figure S24. H&E staining of hearts, livers, spleens, lungs, and kidneys from healthy mice after different treatments for 28 days. Scale bars, 200 μm.

18. References

[1] Y. Huang, X. Ding, Y. Qi, B. Yu, F. J. Xu, *Biomaterials* **2016**, *106*, 134.
[2] F. Liu, S. Sheng, D. Shao, Y. Xiao, Y. Zhong, J. Zhou, C. H. Quek, Y. Wang, Z. Hu, H. Liu, Y. Li, H. Tian, K. W. Leong, X. Chen, *Nano Lett* **2021**, *21* (6), 2461.
[3] K. Mori, M. Yanagita, S. Hasegawa, M. Kubota, M. Yamashita, S. Yamada, M. Kitamura, S. Murakami, *Journal of dental research* **2015**, *94* (8), 1149.
